# Genetic/epigenetic effects in NF1 microdeletion syndrome: beyond the haploinsufficiency, looking at the contribution of not deleted genes

**DOI:** 10.1007/s00439-024-02683-0

**Published:** 2024-06-14

**Authors:** Viviana Tritto, Paola Bettinaglio, Eleonora Mangano, Claudia Cesaretti, Federica Marasca, Chiara Castronovo, Roberta Bordoni, Cristina Battaglia, Veronica Saletti, Valeria Ranzani, Beatrice Bodega, Marica Eoli, Federica Natacci, Paola Riva

**Affiliations:** 1 Department of Medical Biotechnology and Translational Medicine (BIOMETRA), University of Milan, Segrate, Milan Italy; 2 Institute for Biomedical Technologies (ITB), National Research Council (CNR), Segrate (Milan), Italy; 3 Medical Genetics Unit, Woman-Child-Newborn Department, Fondazione IRCCS Ca’ Granda-Ospedale Maggiore Policlinico, Milan, Italy; 4 Genome Biology Unit, Istituto Nazionale di Genetica Molecolare (INGM) “Romeo ed Enrica Invernizzi”, Milan, Italy; 5 Developmental Neurology Unit, Fondazione IRCCS Istituto Neurologico Carlo Besta, Milan, Italy; 6 Department of Biosciences (DBS), University of Milan, Milan, Italy; 7 Molecular Neuroncology Unit, Fondazione IRCCS Istituto Neurologico Carlo Besta, Milan, Italy

## Abstract

NF1 microdeletion syndrome, accounting for 5–11% of NF1 patients, is caused by a deletion in the *NF1* region and it is generally characterized by a severe phenotype. Although 70% of NF1 microdeletion patients presents the same 1.4 Mb type-I deletion, some patients may show additional clinical features. Therefore, the contribution of several pathogenic mechanisms, besides haploinsufficiency of some genes within the deletion interval, is expected and needs to be defined. We investigated an altered expression of deletion flanking genes by qPCR in patients with type-1 NF1 deletion, compared to healthy donors, possibly contributing to the clinical traits of NF1 microdeletion syndrome. In addition, the 1.4-Mb deletion leads to changes in the 3D chromatin structure in the 17q11.2 region. Specifically, this deletion alters DNA-DNA interactions in the regions flanking the breakpoints, as demonstrated by our 4C-seq analysis. This alteration likely causes position effect on the expression of deletion flanking genes.

Interestingly, 4C-seq analysis revealed that in microdeletion patients, an interaction was established between the *RHOT1* promoter and the *SLC6A4* gene, which showed increased expression. We performed NGS on putative modifier genes, and identified two “likely pathogenic” rare variants in RAS pathway, possibly contributing to incidental phenotypic features.

This study provides new insights into understanding the pathogenesis of NF1 microdeletion syndrome and suggests a novel pathomechanism that contributes to the expression phenotype in addition to haploinsufficiency of genes located within the deletion.This is a pivotal approach that can be applied to unravel microdeletion syndromes, improving precision medicine, prognosis and patients’ follow-up.

**Supplementary Information:**

The online version contains supplementary material available at 10.1007/s00439-024-02683-0.

## Introduction

Neurofibromatosis type 1 (NF1) microdeletion syndrome is caused by a heterozygous deletion of 17q11.2 region, encompassing *NF1* gene and accounting for 4.7–11% of patients affected by neurofibromatosis type-I (Kehrer-Sawatzki et al. 2017). NF1 microdeletion patients generally display a more severe phenotype than patients with *NF1* gene mutation, showing variable facial dysmorphism, developmental delay, cognitive impairment, a high number of early-onset neurofibromas, cardiovascular malformations (Venturin 2004), and an increased risk of malignant peripheral nerve sheath tumors (MPNSTs) (De Raedt et al. 2003; Pasmant et al. 2010; Zhang et al. 2015).

Most deletions result by Non-Allelic Homologous Recombination (NAHR) between Low Copy Repeats (LCRs) located centromerically and telomerically *NF1* gene. According to the localization of breakpoints within specific LCRs, three types of deletion can be identified: the 1.4 Mb type-I (70–80%), the 1.2 Mb type-II (10%) and the 1 Mb type-III (1–4%) deletions (Raedt et al. 2006; Pasmant et al. 2010; Messiaen et al. 2011). A small percentage of *NF1* microdeletions (8–10%) are atypical, and they were generally found to be mediated by *Alu* repeats by means of Homologous Recombination Mechanism (HRM), or by other mutational mechanisms including Non Homologous End Join (NHEJ) (Gervasini et al. 2004, 2005) and the SINE/variable number of tandem repeats/*Alu* (SVA) insertion-associated mechanism (Vogt et al. 2014).

NF1 microdeletion patients generally show a severe form of NF1, with a variable expressivity of clinical signs (Mautner et al. 2010). To dissect the causes of variable expressivity of clinical manifestations, the occurrence of different events should be evaluated considering: the *NF1* gene mutation type (Pasmant et al. 2012), the breakpoint location together with the deletion size consistent with the genes included in the deletion interval, and the somatic mosaicism. The last mentioned mechanism is particularly relevant in type-II and atypical deletions (Kehrer-Sawatzki et al. 2004; Vogt et al. 2014) and can have a different impact on the phenotype severity depending on the proportion of normal versus deleted cells and on the cell lineages derived from the cells with microdeletion (Messiaen et al. 2011). Interestingly, the type-I NF1 microdeletion is mostly originated by a germline mutation and represents the largest fraction of the NF1 microdeletion patients, making this cohort the most suitable for genotype-phenotype correlation studies. Co-deletion of different genes pinpoints to possible consequences of haploinsufficiency for some of them as far as the onset of more severe phenotypes in NF1 microdeletion patients: hemizygosity of *RNF135* has been correlated to facial dysmorphisms and/or overgrowth (Douglas et al. 2007; Ferrari et al. 2017), *ADAP2* to cardiovascular malformations (Venturin et al. 2014) and *OMG* to intellectual disability (ID) (Kehrer-Sawatzki et al. 2017). Nevertheless, the evaluation of the phenotypic impact of haploinsufficiency of genes included in the deletion interval by determination of the probability of the Loss-of-Function (LoF) intolerance (pLI) from ExAC data set (Lek et al. 2016) indicates that *RNF135* and *ADAP2* are LoF tolerant and the effect of their hemizygosity should be further investigated. Differently, *SUZ12* is LoF intolerant, it is involved in silencing of many genes (Di Croce and Helin 2013), playing an important role during embryonic development and cancer. Consistently, *SUZ12* is highly expressed during early stage of heart development (Venturin et al. 2005) and its LoF is associated to MPNSTs and in general the early onset of several neurofibromas in NF1 microdeletion patients (Mensink 2005; Pasmant et al. 2010).

Because it is at now unknown if the haploinsufficiency of LoF intolerant genes included in the deletion intervals fully explains the NF1 microdeletion syndrome phenotype, additional mechanisms, little investigated in microdeletion syndromes, such as position effect, could contribute to the onset of specific clinical traits. Indeed, although the pathogenic effect of microdeletions and copy number variations (CNVs) in general has been mainly explained by considering the genes sensitive to dosage alterations, the potential disruption of genome integrity, causing changes in its regulatory architecture, as well as the impairment of gene expression profiles, has been poorly investigated in microdeletions (Amarillo et al. 2013; Lupiáñez et al. 2015; Spielmann et al. 2018; Tritto et al. 2022). The analysis of topologically associating domains (TADs) is important to understand the large-scale functional organization of the regulatory genome of chromosomal regions involved in structural alterations (Rajderkar et al. 2023). Because some TAD structures and enhancer-promoter interactions are conserved across developmental tissues and adult human cells such as fibroblasts or blood cells (Remeseiro et al. 2016), these predictions can be experimentally investigated even if the affected tissue is not available. Furthermore, circular chromosome conformation capture technique followed by sequencing (4C-seq) in patient cells can provide diagnostically valuable information to understand the clinical impact of structural variants by position effect (Gheldof et al. 2013; Laugsch et al. 2019).

Position effect on expression of genes flanking an NF1 deletion has been described for the first time in a patient carrying an atypical NF1 microdeletion (Ferrari et al. 2017). The loss of a chromatin boundary of TADs included in the deletion leads to an aberrant adoption of distal *cis*-acting regulatory elements, causing gene expression deregulation in the regions proximal and distal to the deletion breakpoints. A comparative mapping of TAD alterations in different NF1 microdeletions can shed light on the role of position effect on gene expression deregulation in 17q11.2 region and its impact on phenotype expression.

Although it is known that the majority of patients with type-1 deletions have severe cognitive deficits and developmental delay than patients with intragenic *NF1* mutations, the variability of the clinical phenotype among type-I NF1 microdeletion patients could be addressed by considering the role played by pseudo-dominance and variants in the modifier genes. Pseudo-dominance unmasks recessive variants, randomly occurring in genes within the deletion interval present on the normal chromosome 17 and associated to peculiar traits. This mechanism is underestimated at constitutional level, because the presence of microdeletion mainly addresses the identification of somatic mutations of onco-suppressor genes. Moreover, NF1 is a dominant RAS pathway disorder and a variant in a second gene, encoding an interacting partner or an effector of the same pathway, can worsen the clinical phenotype, contributing to inter-intra-familial variable expressivity (Ferrari et al. 2020; Tritto et al. 2023a). Variants with modifier significance and subclinical effect are generally classified as polymorphisms for their presence in the normal population because they are not subjected to genetic constraint. They can be detected by NGS, even if they are generally not selected by the pipelines commonly applied for identification of pathogenic variants (Deltas 2018). Some glomerulopathies, cystic fibrosis and thalassemias are well studied examples of diseases in which the contribution of secondary functional DNA variants leads to a configuration of the final phenotype (Gallati 2014; Deltas 2018; Mettananda and Higgs 2018).

Given the highest prevalence of type-I NF1 microdeletion, among the NF1 microdeletion patients, we enrolled 22 patients, studied their clinical phenotype, and evaluated the effect of their microdeletion dissecting the role of position effect, pseudo-dominance and modifier gene variants to provide new insights into identifying the pathogenesis of NF1 microdeletion syndrome. This is a paradigmatic study aimed at increasing knowledge on the etiology of microdeletions syndromes. These results provide new insights to address genotype-phenotype correlation with a positive impact on patient’s management and future development of druggable targets and the effective pharmacological therapies.

## Materials and methods

### Patients

Eligible patients were identified by scanning the electronic NF1 patient database at Fondazione IRCCS C. Besta and Fondazione IRCCS Cà Granda Ospedale Maggiore Policlinico. All the clinical features were derived from the medical information collected during the clinical follow-up of the eligible patients. Medical records were surveyed, the following data were collected at the time of mutation analysis and re-verified for accuracy at the time of this study: data of birth, gender, age at the time of genetic testing, mode of inheritance, NF1 signs and symptoms including visual impairment, pain, epilepsy, cognitive impairment, plexiform, optic nerve glioma (OPG), other neoplasms of central nervous system (CNS) and of other organs.

Although the clinical information obtained is in-depth, it lacks precise standardization regarding the degree of intellectual involvement and the real tumor burden; in fact, not all patients have undergone cognitive tests or whole-body magnetic resonance imaging (MRI). The presence of cognitive impairment was attested by cognitive test assessment or suspected by clinical evaluation while the presence of internal neurofibromas was established on the basis of different imaging studies (Abdominal Ultrasound/ Whole-body MRI, Thorax or Abdominal MRI), when available. All the clinical information was obtained thank to the clinical follow-up; the follow-up management follows the current guidelines of Regione Lombardia dedicated to NF1 patients (Diagnostic, Therapeutic and Care Pathways (PDTA) http://malattierare.marionegri.it/images/downloads/PDTA/PDTA_schede/nf1.pdf).

A total of twenty-two patients satisfied the inclusion criteria, which included the presence of type-I NF1 microdeletion, previously characterized by MLPA or Array CGH analysis, and were enrolled for the study.

### Prediction of TADs and regulatory elements

All predictions of TADs and regulatory elements mapping in the 17q11.2 region, including the type-I *NF1* microdeletion and the flanking region, were performed by setting the Human GRCh37/hg19 build as reference assembly. The chromosomal coordinates chr17:26646000–32484000 were chosen for the region to be investigated, according to the extension of the genomic region including the 17q11.2 genes designed for our Target Resequencing panel. The breakpoints of the type-I *NF1* microdeletion were set at positions chr17:28995000 and chr17:30411500, according to the localization of the PRS2 paralogous recombination sites, where recombination events that determine the type-I *NF1* microdeletion occur with greater frequency (Summerer et al. 2018).

We interrogated the 3D Genome Browser (http://3dgenome.fsm.northwestern.edu/) to view the Hi-C interactions, which mirror TADs, in GM12878, NHEK, DLPFC and GZ cell lines. VISTA Enhancer Browser (https://enhancer.lbl.gov/) and CTCFBSDB 2.0 (http://insulatordb.uthsc.edu/) were interrogated to identify the regulatory elements (enhancer and insulators, respectively) predicted to be included within the type-I *NF1* microdeletion and in the flanking region.

In order to visualize the genes and the regulatory elements included in the region of interest simultaneously, on the UCSC Genome Browser (https://genome.ucsc.edu/), we selected the “UCSC Genes” and added two custom tracks, one for VISTA Enhancer and the other one for the CTCF binding sites previously identified (Supplementary Table S1).

### Reverse transcription (RT) and quantitative real-time PCR (qPCR)

Total RNA (500 ng) was reverse transcribed using the iScriptTM cDNA Synthesis Kit (Bio-Rad Laboratories Inc., Barkeley, CA, USA). Twelve 17q11.2 genes included in our Target Resequencing panel and reported to be expressed in whole blood in GTEx portal (https://gtexportal.org) were selected for the study: eight out of 50 genes mapping in the centromeric region to the type-I *NF1* microdeletion (*IFT20*, *PHF12*, *ABHD15*, *SSH2*, *NSRP1*, *BLMH*, *CPD*, *GOSR1*) and four out of 10 genes of the telomeric region (*RHOT1*, *c17orf75*, *ZNF207*, and *PSMD11*). Furthermore, *SLC6A4* and six genes included in the type-I *NF1* microdeletion interval and expressed in whole blood (*CRLF3*, *NF1*, *EVI2B*, *EVI2A*, *COPRS*, *UTP6*) were later selected following the 4C-seq assay results. *EIF4A2* gene was used as a housekeeping control for normalization. The genomic locations of the selected genes and the specific oligonucleotides for qPCR assays are shown in Supplementary (Supplementary Tables S2 and S3, respectively). Each SYBR Green qPCR assay was performed using GoTaq–qPCR master mix (Promega) and run on a QuantStudio 5 Real-Time PCR Systems (Thermo Fisher Scientific).

### Statistical analysis

All qPCR experiments were run in triplicate and the average of the threshold cycles (Ct) for each sample was made. To determine the relative gene expression, the 2^−ΔCt^ method was applied (ΔCt = Ct gene target – Ct housekeeping gene, for each sample). For each gene analyzed, mean, standard deviation, standard error of the mean, and confidence intervals values were calculated in the two groups of samples, which include 15 patients with type-I NF1 microdeletion and 15 healthy donors, and the two-tailed Student’s *t*-test with equal variance was applied to compare the means, after excluding outliers identified by the Tukey test (Supplementary Table S4). The *p*-values were corrected by the Benjamini–Hochberg (BH) method and the results were considered statistically significant when BH-adjusted *p* < 0.05. Statistical analysis was performed using Statistics Kingdom software (https://www.statskingdom.com/).

### Circular chromosome conformation capture followed by sequencing (4C-seq)

The 4C-seq assay was carried out on peripheral blood mononuclear cells (PBMCs) (5 × 10^6^ cells) from three patients with type-I NF1 microdeletion (N44, N83 and N86) and three healthy donors. The promoter of *RHOT1*, the first gene mapped after the telomeric deletion breakpoint, was chosen as a viewpoint. 4C-seq was performed according to the protocol previously described by Krijger et al. (Krijger et al. 2020), to which the following modifications were applied: the first restriction enzyme digestion, first ligation, second restriction enzyme digestion, and second ligation were performed using the enzymes DpnII (150U, New England BioLabs), T4 DNA Ligase (200U, New England BioLabs), HindIII (3 rounds of 200U, New England BioLabs), and T4 DNA Ligase (100U), respectively; 3 C template and 4C template were purified by phenol-chloroform method. The 4C-seq libraries were generated from 200 ng of 4C template through two PCR steps, using Phusion High-Fidelity PCR Kit (ThermoFisher) and 0.5 µM of primers (Supplementary Table S5). The thermal cycling conditions for the first amplification were 98 °C for 1 min, 16 cycles of 98 °C for 20 s, 52.5 °C for 45 s and 72 °C for 2.5 min, a final elongation at 72 °C for 5 min. The thermal cycling conditions for the second amplification were 98 °C for 1 min, 20 cycles of 98 °C for 20 s, 60 °C for 45 s and 72 °C for 2.5 min, a final elongation at 72 °C for 5 min. 4C-seq libraries were sequenced in single end modality with a read length of 120 and 150 bp on the Illumina NextSeq 500 platform.

### 4C-seq data analysis

The 4C-seq data analysis was performed according to the pipeline previously described by Krijger [35] (https://github.com/deLaatLab/pipe4C v. 1.1.4’). The reads obtained from the sequencing of the three healthy donors and three patients with type-I NF1 microdeletion were collected into two pools (HD_pool and DEL_NF1_pool), obtaining 15 M and 20 M respectively. To identify preferential chromosome regions of interaction with the 4C viewpoint, the peak calling analysis was carried out using the PeakC tool (https://github.com/deWitLab/peakC) [36] using the following parameters: qWr = 2.5, wSize = 21, minDist = 20,000, alphaFDR = 0.001. The same was performed on each sample individually (qWr = 1, wSize = 21, minDist = 20,000, alphaFDR = 0.001). The called peaks were annotated according to human reference annotation (GENCODE v.38) considering both gene locus and 2.5 kb upstream (promoter) using BEDTools v.2.29.2 (Quinlan and Hall 2010). Enhancer analysis was performed by downloading a ChromHMM 18-state model of PBMCs from the ENCODE database (ENCSR852VXN); then the genomic coordinates of chromatin states related to enhancers (EnhA1, EnhA2, EnhBiv, EnhG1, EnhG2, EnhWk) were considered and intersected with the called peaks using BEDTools.

### NGS analysis

To identify the pathogenic variants associated with the clinical condition of the enrolled patients, we designed a Target Resequencing panel including the *NF1* gene and 16 of its direct interactors, 49 genes belonging to the RAS/MAPK pathway, 13 additional protein-coding gene located in the microdeletion 17q11.2, and 60 genes in its flanking regions, for a total of 139 genes (Supplementary Table S6). The enrichment of regions of interest was performed using Agilent SureSelect XT technology (www.agilent.com). The probes design was carried out to include the 5’UTR, the 3’UTR and the protein-coding regions of all selected genes. Target resequencing was performed on blood samples of 19 patients by pooling all indexed samples in a single run (2 × 300 bp, 600 cycles) of Illumina MiSeq platform. Parents were not available for the analysis.

Read quality assessment and trimming for 200 bp length were performed using FastQC (v. 0.11.8; http://www.bioinformatics.babraham.ac.uk/projects/fastqc/) and Trimmomatic (v. 0.36) (Bolger et al. 2014), respectively. Then, the QC-checked paired end (PE) reads of each sample were mapped to the NCBI human reference genome (build GRCh37) using BWA-MEM aligner (0.7.10-r789) (Li 2013). Mapping was done performed allowing for a maximum 3 mismatches and using other default parameters of BWA. We then used samtools (Li et al. 2009) to remove the duplicate reads due to PCR amplification during library preparation. For each sample, we retain only high quality (HQ) alignments in sorted BAM files (HQ-BAM) by filtering out unmapped reads and those alignments with mapping quality (MAPQ) less than 15. These high-quality alignments (HQ-BAMs) are then checked for overall mapping statistics (mapping-QC) by an in-house script. The detailed mapping statistics for each sample is reported in Supplementary Table S7.

GATK software (v. 3.4) (DePristo et al. 2011) was then used to perform quality score recalibration (using the TableRecalibration walker), local realignment around known indels (using the IndelRealigner walker) and variant calling (by the HaplotypeCaller walker) for both single nucleotide variants (SNVs) and insertions/deletions (indels). Poorly confident variants having QUAL < 150, Fisher Strand (FS) strand bias > 60 for SNV and > 200 for indels, or three SNVs within 10 base-windows were flagged for removal in the FILTER field of the VCF file.

### Variant analysis and interpretation

Functional annotation and impact effect prediction were performed using ANNOVAR (v. 20191024) (Wang et al. 2010), which includes prediction scores from 31 prediction algorithms and 8 conservation scores from the dbNSFP database (https://sites.google.com/site/jpopgen/dbNSFP). To predict changes in protein stability upon point mutation, the Elaspic (Witvliet et al. 2016) and DynaMut tools (Rodrigues et al. 2018) were used.

In addition, for each gene constraint metrics parameters as reported in the gnomAD v.2.1.1 database (https://gnomad.broadinstitute.org/) (Karczewski et al. 2020), namely, pLI, pRec, and pNull for loss of function variants, and *Z*-score for missense variants (Lek et al. 2016), were considered. Accordingly, genes with a pLI ≥ 0.9 were classified as haploinsufficient, whereas genes with a missense *Z*-score ≥ 3.09 were considered to be significantly intolerant to heterozygous missense variants.

Variants with a MAF < 0.01 according to both 1000 Genomes database (https://www.internationalgenome.org/, release 20130502) (Auton et al. 2015) and gnomAD v.2.1.1 were considered rare. In addition, SNVs not reported neither in public databases, such as 1000 Genomes Project, gnomAD v.2.1.1, dbSNP (https://www.ncbi.nlm.nih.gov/snp/, Build 154, April21 2020) (Sherry 2001), DECIPHER v.11.0 (https://decipher.sanger.ac.uk/) (Firth et al. 2009), and ClinVar (https://www.ncbi.nlm.nih.gov/clinvar/, last access December 23 2020) (Landrum et al. 2018), nor in PubMed (https://pubmed.ncbi.nlm.nih.gov/, last access December 23 2020) were classified as novel.

We then manually assessed the clinical significance of the SNVs according to the American College of Medical Genetics (ACMG)/Association of Molecular Pathology (AMP) guidelines (Richards et al. 2015), taking into account the novelty of the variant, possible associations of the affected genes with mendelian disorders according to the OMIM (Online Mendelian Inheritance in Man) database (https://omim.org/, last access December 23 2020) (Hamosh 2004), previous inclusion in databases such as DECIPHER v.11.0, ClinVar, and COSMIC (Catalogue of Somatic Mutations in Cancer) v.92 (https://cancer.sanger.ac.uk/cosmic) (Tate et al. 2019), and/or in PubMed, localization of the variant in functional domains that could be mutational hotspots, *in silico* prediction of pathogenicity based on conservation and type of amino acid substitution, and constraint metrics.

Specifically, we classified the variant into three groups: (1) “Likely benign”, when more than one concordant benignity criterium was present; (2) “Uncertain”, when a few supporting evidence of both pathogenicity and benignity was present; (3) “Likely pathogenic”, when evidence supporting pathogenicity were concordant among a number of different *in silico* predictors although at least one major pathogenicity criterium, such as either detection in other patients with similar phenotypes or variant functional validation, was still missing.

## Results

### Patients’ description

Our cohort consisted of 22 NF1 microdeletion patients (12 males and 10 females), with a mean age at last evaluation of 31 years (7 pediatric and 15 adult cases). All patients bear a type-I NF1 microdeletion syndrome, characterized by MLPA or array CGH analysis, and fulfil the NIH diagnostic criteria for NF1, with at least two of the major diagnostic criteria. Complete clinical data are not available for patients N84 and N85. Cafè au lait spots (CALs) are described in almost all the patients (21/22), while folds or cutaneous freckling in 15. Two (or more) cutaneous or plexiform neurofibromas are present in 16, OPG in only one, and Lisch nodules in 14. No patients present with osseous dysplasia. Regarding the presence of neurofibromas, cutaneous ones were present is 16 patients. Among them, 10 patients present at least with a plexiform neurofibroma (Table 1).

**Table 1:** Election diagnostic criteria of type-I NF1 microdeletion patients

Patient ID	Sex	Age at last evaluation (years)	CALs (HP: 0007565)	Axillary/ groin freckling (HP:0001480)	Number of cutaneous nf /plexiform nf (HP:0009732)	OPG (HP:0009734)	> 2 Lisch nodules (HP:0009737)
N20	F	44	+	+	###/2	-	+
N21	F	35	+	+	#/2	-	+
N22	M	29	+	+	#/1	-	+
N26	M	45	+	-	##/-	-	+
N27	M	42	+	+	###/1	-	-
N28	M	30	+	+	##/2	-	+
N43	F	47	+	+	###/-	-	+
N44	F	32	+	+	##/2	-	+
N45	F	39	+	+	##/1	-	+
N75	M	10	+	-	-/-	-	-
N76	F	16	+	+	-/-	-	+
N77	F	17	+	+	##/1	+	+
N78	M	25	+	-	###/1	-	+
N79*	F	10	+	+	-/-	-	-
N80	F	71	+	+	###/-	-	+
N81	M	27	+	-	#/-	-	-
N82	M	38	+	-	###/1	-	+
N83	M	30	+	+	##/-	-	+
N84*	M	NA	+	NA	NA	NA	NA
N85*	M	NA	+	+	-/-	NA	NA
N86	F	37	-	+	#/-	-	-
N87	M	5	+	-	-/-	-	-

CALs, Café Au Lait macules; F, female; M, male; NA, not available; nf, neurofibromas; OPG, Optic Pathway Glioma; +, present; -, absent; # = 1–10 cutaneous neurofibromas; ## = 11–100 cutaneous neurofibromas; ### = >100 cutaneous neurofibromas; *= included in the effect position study and absent in the NGS study patients. Whenever possible, for each of the clinical features the corresponding item in the HPO (Human Phenotype Ontology) phenotype vocabulary has been reported within brackets

Subcutaneous neurofibromas are reported in 14 patients; spinal neurofibromas in 6. Cervical, brachial or lumbosacral plexus neurofibromas are described in 4 patients, while a mediastinic or abdominal localization in 3. MPNSTs were diagnosed in 3 patients, while other two patients experienced a CNS glioma. No other NF1 related tumor (GIST, NET or pheochromocytoma) was diagnosed. Information on the presence of internal tumors is not available in patients N84, N85, and N86, as they were not examined by whole-body MRI. Intellectual disabilities (attested by cognitive test assessment or suspected by clinical evaluation) are described in the majority of patients (16/20), epilepsy was diagnosed in 2, brain MRI demonstrated Unidentified Bright objects (UBOs) in 11 patients. Cerebrovascular anomalies were found in 3 patients, while no other NF1 associated vascular dysplasia were diagnosed. Typical findings in NF1 microdeletion patients like overgrowth and dysmorphisms are present in 5 and 18 patients, respectively. Pectus esxcavatum and motor delay are reported in one patient. Owing to NF1 routine clinical/instrumental follow-up the following complications were diagnosed: hypertension in two patients, Chiari type 1 malformation, thyroid C-Cell Hyperplasia, adrenal ganglioneuroma and diaphragmatic relaxation in three different patients. In one patient congenital hypothyroidism and facial hemangiomas were present in the first infancy (Table 2).

**Table 2:** Clinical features of type-I NF1 microdeletion patients

Ref.	Sex	Sub cutaneous nf (HP:0100698)	Spinal nf (HP:0009735)	Internal nf (localization)	MPNST (HP:0100697)	Other tumors	Scoliosis (HP:0002650)	Vascular abn. (HP:0002597)	ID (HP:0001249)	Overgrowth (HP:0001548)	Dysmorphic features (including coarse facial features) (HP:0001999)	Other
N20	F	-	-	+ (cervical and sacral plexus; mediastinum)	-	-	-	-	+	- (big hands and feet)	+	-
N21	F	-	MNFSR	-	+	-	-	-	+	-	-	UBOs, Chiari type I malformation
N22	M	+	MNFSR	-	-	-	+	-	+	+	+	UBOs
N26	M	+	-	-	-	-	-	-	+	+	+	UBOs
N27	M	+	-	+ (brachial plexus)	+	-	-	-	+	+	+	Hypertension
N28	M	+	-	-	-	-	-	-	+	+	+	-
N43	F	+	-	-	-	-	-	+ (cerebro vascular)	+	-	+	UBOs; thyroid C-Cell Hyperplasia
N44	F	-	SNF	-	-	-	-	-	+	- (big hands and feet)	+	UBOs, adrenal ganglio neuroma, diaphragmatic eventration;
N45	F	+	-	-	-	-	-	-	+	-	+	-
N75	M	+	NA	-	-	+ (brain stem glioma)	-	-	-	-	+	UBOs; congenital hypothyroidism; two facial hemangiomas
N76	F	-	-	-	-	-	-	-	+	-	+	UBOs
N77	F	+	-	-	-	+ (brain stem glioma)	-	-	+	-	+	UBOs
N78	M	+	SNF	+ (lumbar plexus, mediastinum, abdomen)	-	-	+	+ (cerebro vascular)	-	-	+	Epilepsy; UBOs
N79*	F	+	MNFSR	-	-	-	+	-	-	-	+	UBOs, pectus esxcavatum, motor delay
N80	F	+	-	-	-	-	NA	-	-	-	+	Hypertension
N81	M	+	-	-	-	-	-	-	+	-	+	-
N82	M	+	-	+ (lumbar plexus)	-	-	-	-	+	-	+	-
N83	M	+	SNF	+ (mediastinum, abdomen)	+	-	+	-	+	+	+	-
N84*	M	NA	NA	NA^#^	NA	NA	NA	NA	NA	NA	NA	NA
N85*	M	-	NA	NA^#^	NA	NA	-	NA	NA	NA	NA	NA
N86	F	-	-	NA^#^	-	-	-	+ (cerebro vascular)	+	-	+	Epilepsy
N87	M	-	NA	-	-	-	-	-	+	-	-	UBOs

Nf = Neurofibromas

SNF = spinal neurofibromatosis involving all the nerve roots

MNFSR = multiple spinal neurofibromas involving some but not all the nerve roots

NA = data not available

^#^ = whole-body MRI not performed

MPNST = Malignant Peripheral Nerve Sheath Tumors

Vascular abn. = vascular abnormalities (cerebrovascular abnormalities, aneurysms, renal artery stenosis)

ID = Intellectual Disability

UBOs = Unidentified Bright Objects

*= included in the effect position study and absent in the NGS study patients

Whenever possible, for each of the clinical feature within the brackets has been reported the corresponding item in the HPO (Human Phenotype Ontology) phenotype vocabulary

### Pathogenetic role of epigenetic alterations in the NF1 microdeletion syndrome

To evaluate whether epigenetic mechanisms may contribute to the severity of the clinical phenotype of patients carrying NF1 microdeletions, the regulatory landscape of the 17q11.2 region involved by the deletion and the position effect on genes flanking the type-I NF1 microdeletion were investigated. In addition, the role of the type-I NF1 microdeletion on the occurrence of position effect was further detailed using 4C-seq assays.

### In silico analysis of 17q11.2 region’s regulatory landscape

**Fig. 1:**
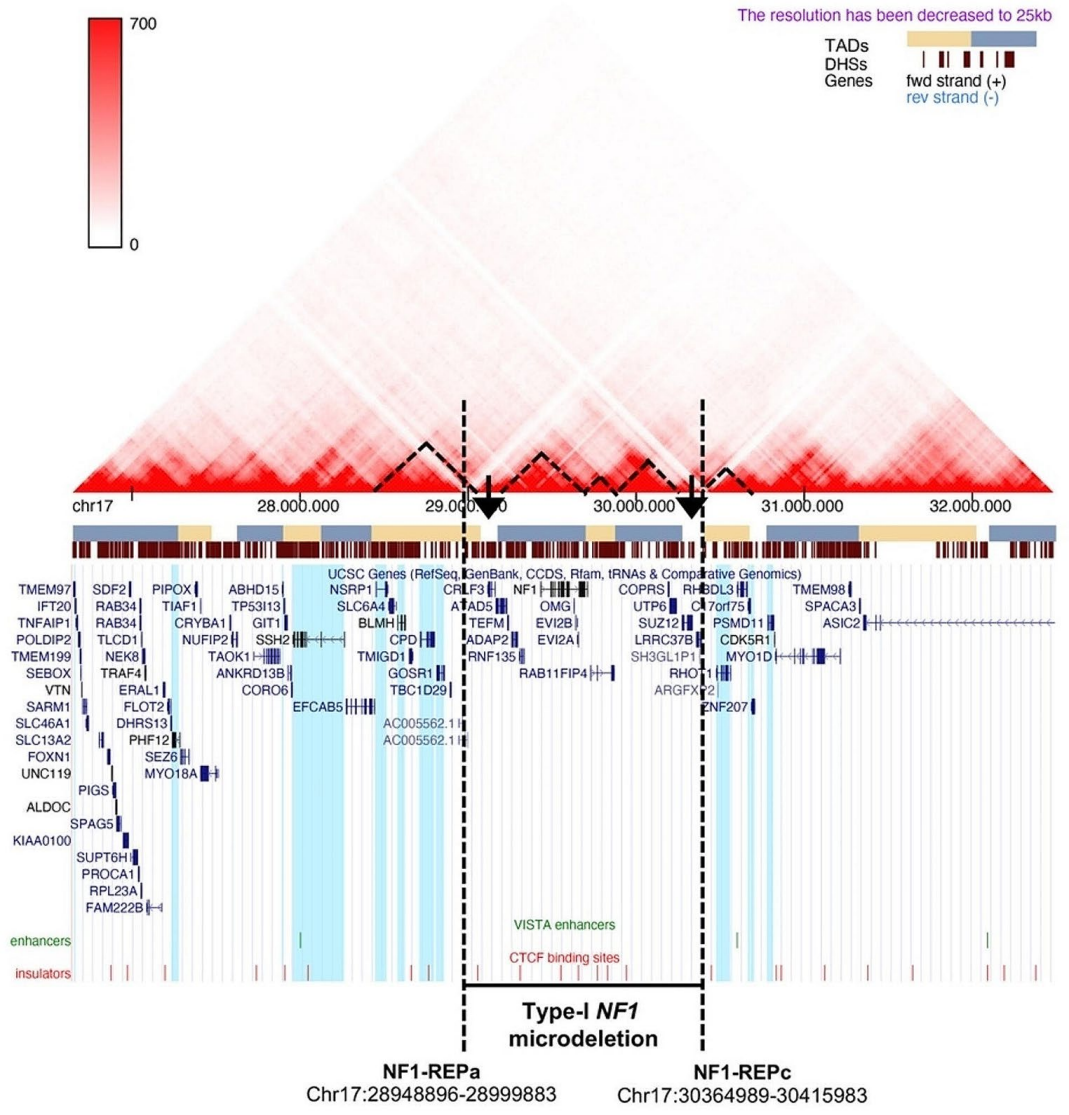
Visualization of the topologically associating domains (TAD) mapping in the 17q11.2 genomic region. The heatmap obtained from 3D Genome Browser shows the TADs (highlighted by black triangles) mapping in the 17q11.2 region, whose position along the chromosome is indicated by the yellow and cerulean blue bars, in accordance with the Hi-C data in blood-derived GM12878 lymphoblastoid cells. The brown barcode represents the DNase I hypersensitive sites (DHSs). The UCSC Genome Browser screen displays genes (in blue-black), enhancers (in green) and insulators (in red) for the same region. The figure also shows the type-I NF1 microdeletion and the coordinates of the corresponding breakpoints. The genes, whose expression levels were evaluated by qPCR, are highlighted in light blue. The type-I NF1 microdeletion removes two extended boundaries between TADs (black arrows), part of one TAD and three whole TADs including 14 protein-coding genes and 7 predicted insulators

To evaluate a possible position effect on the expression regulation of the genes flanking the type-I NF1 microdeletion, we investigated the topological domain landscape in the chromosomal region including the deletion, in blood-derived GM12878 lymphoblastoid cells. As shown in Fig. 1, type-I NF1 microdeletion involves four TADs: one is only partially deleted, while three are totally removed. In addition, the deletion eliminates two extended genomic boundaries between TADs and could alter the activity of *cis*-regulatory elements, such as enhancers, silencers and insulators, impairing the expression of genes flanking the deletion. *In silico* predictions of the regulatory elements showed that although there were no enhancers within the deletion, three enhancers were located in the flanking region, one of which was only 177 kb from the telomeric breakpoint of the deletion within the first TAD not deleted. Furthermore, twenty-four insulators were predicted in the chromosomal region investigated and seven out of them were found within the deletion. The IDs and the genomic coordinates of the identified regulatory elements are shown in Supplementary (Supplementary Table S1).

### Evaluation of position effect on genes flanking type-I NF1 microdeletion

**Fig. 2:**
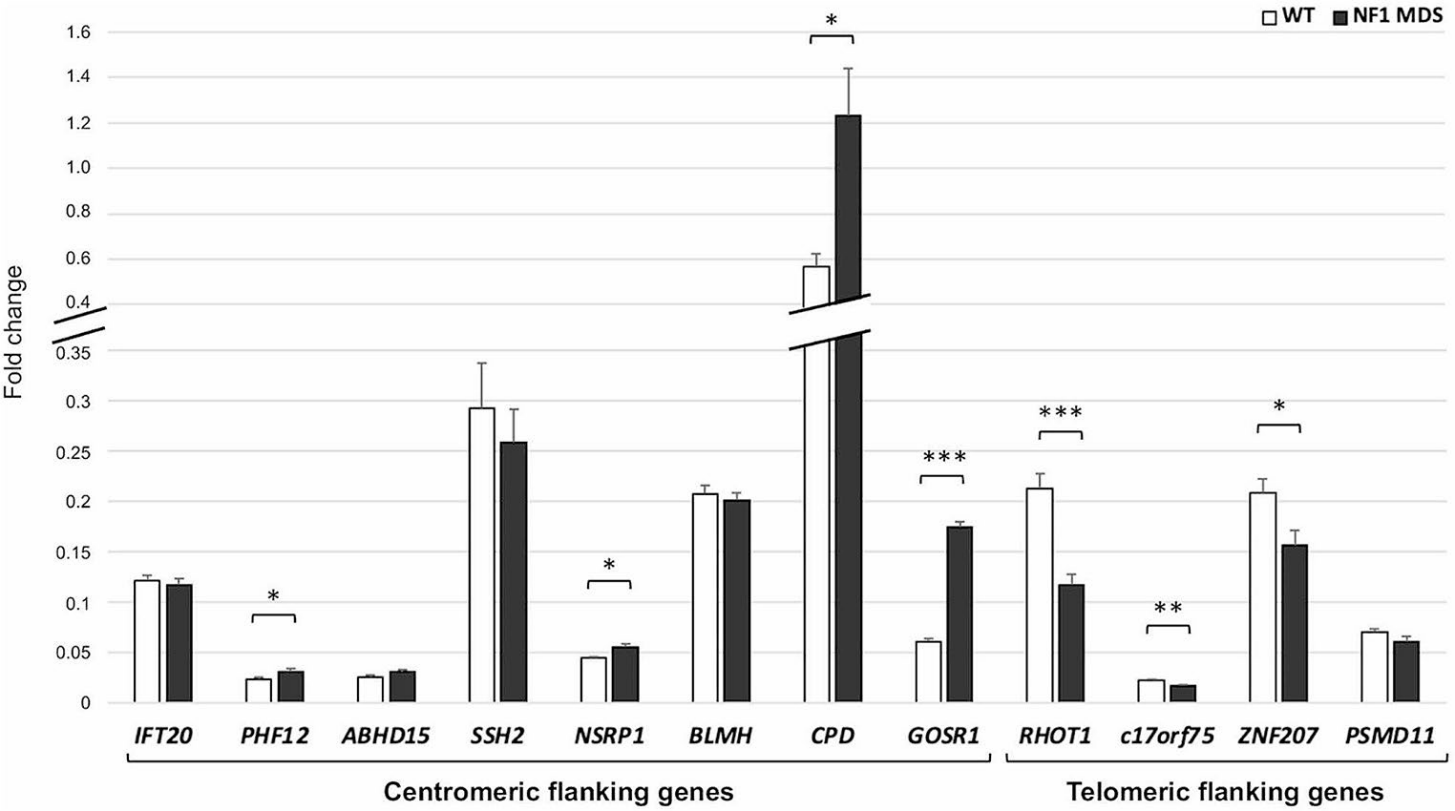
Gene expression analysis of the selected genes flanking the type-I NF1 microdeletion. The histogram shows the mean of the quantitative expression levels (2^−ΔCt^) in peripheral blood from 15 patients with type-I NF1 microdeletion syndrome (NF1 MDS, shown in black) and 15 wild-type controls (WT, shown in white) of 8 centromeric genes (*IFT20*, *PHF12*, *ABHD15, SSH2*, *NSRP1*, *BLMH*, *CPD*, and *GOSR1*) and 4 telomeric genes (*RHOT1, c17orf75*, *ZNF207*, and *PSMD11*) of type-I NF1 microdeletion. A broken scale is used for the Y-axis. The genes *PHF12*, *NSRP1*, *CPD*, and *GOSR1* were statistically significantly hyper-expressed, whereas the genes *RHOT1*, *c17orf75* and *ZNF207* were statistically significantly hypo-expressed, in NF1 microdeletion patients compared to controls. The other five genes analyzed (*IFT20*, *ABHD15, SSH2*, *BLMH*, and *PSMD11*) showed statistically comparable expression between the two samples’ groups. *n* = 15, mean ± standard error of the mean, * BH-adjusted *p* < 0.05, ** BH-adjusted *p* < 0.01, *** BH-adjusted *p* < 0.001, Student’s *t*-test

To verify our hypothesis of the position effect on the expression regulation of the genes flanking the type-I NF1 microdeletion, we investigated the occurrence of a common aberrant expression profile of genes flanking the deletion in our cohort of patients. We performed a gene expression analysis by qRT-PCR on RNA extracted from peripheral blood comparing in a subset of 15 type-I NF1 microdeletion patients, for whom the RNA was available, versus 15 wild-type controls. For qPCR assays we selected 12 genes that were ubiquitously expressed with expression levels in whole blood greater than 1 TPM (transcripts per million), mapped in TADs close to the deletion up to 2 Mb away from the breakpoints and, where possible, genes in which rare variants had been identified. A statistically significant difference in the average value of the quantitative expression levels (2^−ΔCt^) of seven out of twelve genes analyzed was found between NF1 microdeletion patients and controls, applying the Student’s *t*-test (Supplementary Table S4). As shown in Fig. 2 and Supplementary Fig. S1, four genes (*PHF12*, *NSRP1*, *CPD*, and *GOSR1*) were over-expressed and three genes (*RHOT1*, *c17orf75* and *ZNF207*) were hypo-expressed in NF1 microdeletion patients compared to controls, with a statistical significance of *p* < 0.05, while for the other five genes (*IFT20*, *ABHD15, SSH2*, *BLMH*, and *PSMD11*) a statistically comparable expression level was found between the two samples’ groups (Supplementary Table S4). *SSH2* and *CPD* genes show a marked variability of the specific gene transcript level, with a standard deviation of the average level of gene expression greater than 0.1 (Supplementary Table S4 and Fig. S1), suggesting that other mechanisms contribute, at different levels, to gene expression regulation. On the basis of the gene expression profiles, our results are consistent with a position effect of the NF1 microdeletion in the peripheral blood of patients. Interestingly, the deregulated expression genes that appear to be affected by the position effect are mapped adjacent to the deletion, in the two TADs immediately flanking the type-I NF1 microdeletion, except for the *PHF12* gene (Fig. 1).

### Analysis of three-dimensional chromatin structure impairment by 4C-seq assay

**Fig. 3:**
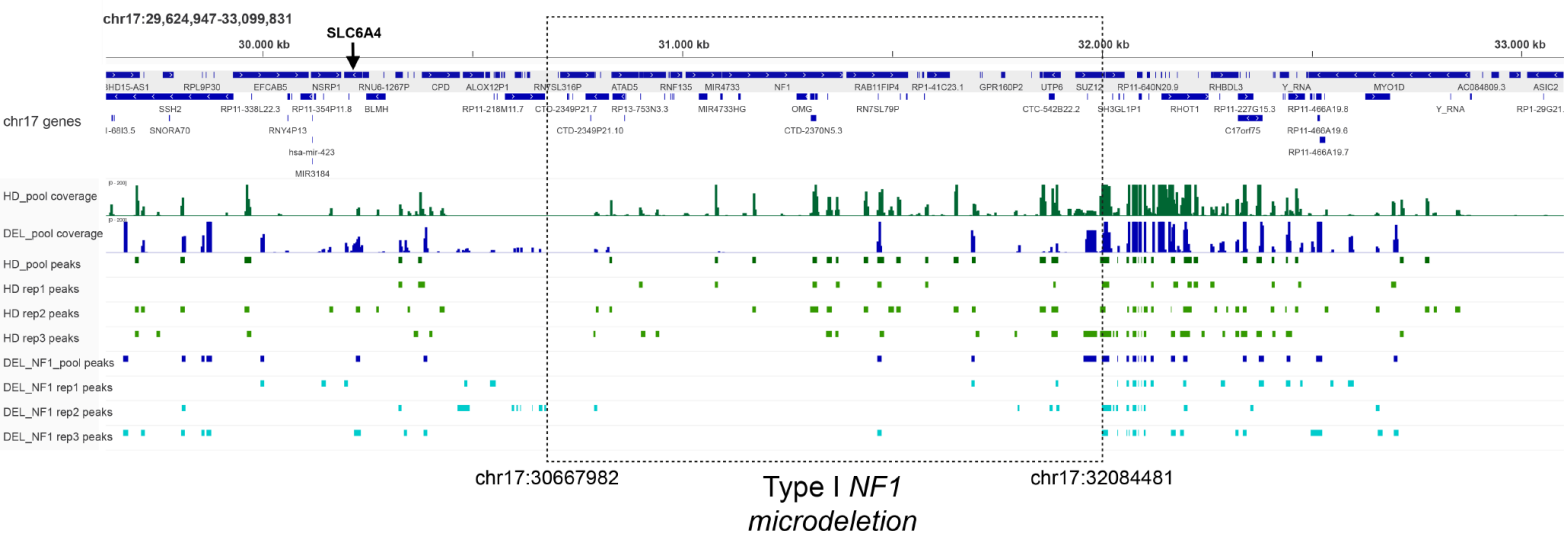
Annotation of peaks identified by 4C-seq data analysis. The figure shows the 4 C-seq peaks mapped to genes in the healthy donor pool (HD_pool) and in the type-I NF1 microdeletion patient pool (DEL_NF1_pool), and in the single samples of each one (rep1, rep2, and rep3). The lack of interactions between the 4C-seq viewpoint and the DNA regions included in the deletion interval is clearly evident in all three microdeletion patient samples. The dashed lines indicate the type-I NF1 microdeletion breakpoints, whose chromosomal coordinates are shown (build Human GRCh38/hg38)

To verify the possible alteration of three-dimensional chromatin structure, which could confirm the role of the 17q11.2 microdeletion in the position effect observed in the peripheral blood of patients with NF1 microdeletion syndrome, a 4C-seq assay was set up. 4C-seq was carried out on PBMCs isolated from three out of 22 patients carrying type-I NF1 microdeletion included in our cohort, and from three healthy donors. The promoter of *RHOT1* was chosen as the 4C viewpoint because it is the first gene mapped after the telomeric deletion breakpoint and belonging to the group of genes susceptible to the position effect (Figs. 1 and 2). Coverage profiles derived by collecting into two pools, the patient pool and the healthy donor pool, the reads obtained by NGS sequencing of each sample, mapping to the 3 Mb-regions upstream and downstream of the viewpoint, are shown in Supplementary Fig. S2. The genes precise chromosomal regions interacting with the viewpoint were identified for both pools of NF1 patients and healthy donors (see Methods and Supplementary Table S8): 21 genes for the healthy donor pool and 12 genes for the NF1 microdeletion syndrome patient pool. NF1 patients shares 11 genes with the healthy donors, while 10 genes are called only in the control pool and one (*SLC6A4*) exclusively in the patient pool. Thus, in patients with type-I NF1 microdeletion a loss of interactions between the *RHOT1* promoter and several genes occurs (Fig. 3).

**Fig. 4:**
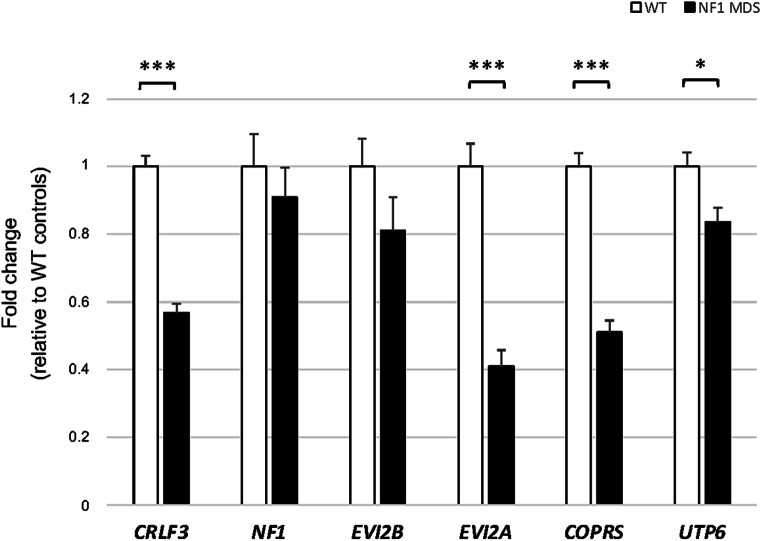
Gene expression analysis of selected genes included in the type-I NF1 microdeletion interval. The histogram shows the mean of the quantitative expression levels (2^−ΔCt^), relative to that of the wild-type controls, of six genes included in the type-I NF1 microdeletion in peripheral blood from 15 patients with type-I NF1 microdeletion syndrome (NF1 MDS, shown in black) and 15 wild-type controls (WT, shown in white). The gene expression levels range from 41 to 91% in patients as compared to healthy donors. Although all of these genes are in a hemizygous state in NF1 microdeletion patients, two genes (*NF1* and *EVI2B*) showed statistically comparable expression between the two samples’ groups. *n* = 15, mean ± standard error of the mean, * BH-adjusted *p* < 0.05, *** BH-adjusted *p* < 0.001, Student’s *t*-test

Interestingly, seven out of ten interactions lost by the patients involve genes mapped to the chromosomal region within the deletion interval (*CRLF3*, *ATAD5*, *NF1*, *EVI2B*, *EVI2A*, *COPRS*, *UTP6*), while one gene interaction is lacking in the chromosome region upstream of the deletion (*TMIGD1*) and two in the chromosome region downstream of the deletion (*ASIC2* and *CCL8*) (Supplementary Table S8). To evaluate possible changes in the expression of genes within the deletion interval that lost interactions with the *RHOT1* promoter, qPCR assays were carried out, on cDNA retrotranscribed from RNA extracted from PBMCs of 15 type-I NF1 microdeletion patients and 15 controls. The qPCR results showed that the expression levels of genes included in the type-I NF1 microdeletion interval in patients ranged from 41 to 91% compared to the expression levels of healthy donors (Fig. 4 and Supplementary Table S9).

Among the genes that lost interaction with the *RHOT1* promoter, *ATAD5*, included in the type-I NF1 microdeletion interval, and three genes flanking the deletion (*TMIGD1*, *ASIC2* and *CCL8*), which are not expressed in blood, were not tested by qPCR.

Interestingly, the peak annotation analysis revealed that the microdeletion patient pool acquired a new DNA-DNA interaction compared with controls, specifically between the *RHOT1* promoter and the *SLC6A4* gene. *SLC6A4* is mapped 450 kb upstream from the centromeric breakpoint of type-I NF1 microdeletion, in the partially deleted TAD. This finding was also confirmed by the peak annotation analysis carried out on the single samples of the pool in two out of three samples. We investigated any possible change in *SLC6A4* transcriptional expression in the NF1 patients, that could be possibly due to changes in chromatin organization due to the microdeletion. We performed qPCR of the 15 type-I NF1 microdeletion patients and 15 controls showing that *SLC6A4* is statistically significantly over-expressed (*p* < 0.001) in the peripheral blood of NF1 microdeletion patients compared to controls (Fig. 4 5 and Supplementary Fig. S3 and Table S10), indicating an altered regulation of *SLC6A4* gene expression, due to chromatin remodeling.

**Fig. 5:**
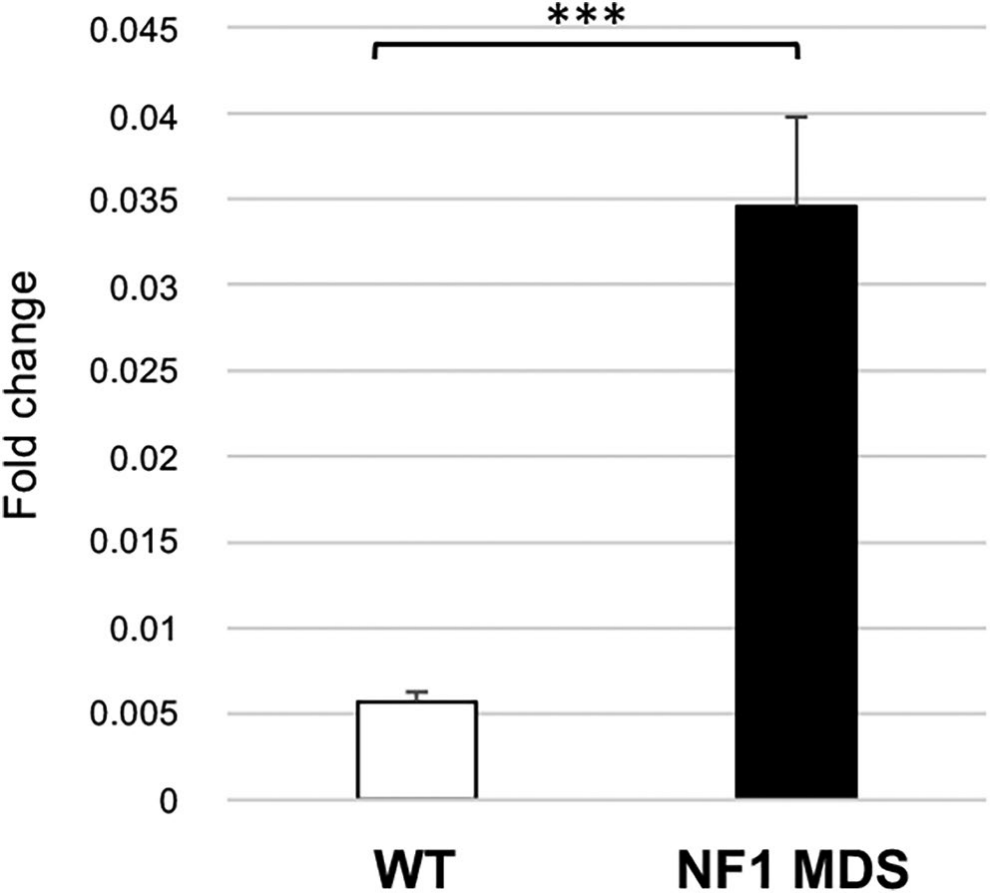
*SLC6A4* gene expression analysis. The histogram shows the average expression level (2^-ΔCt^) of the *SLC6A4* gene in the peripheral blood of 15 patients with type-I NF1 microdeletion syndrome (NF1 MDS, in black) and 15 wild-type controls (WT, in white). The gene was statistically significantly hyper-expressed in NF1 microdeletion patients compared with controls. Mean ± standard error of the mean, Student’s *t*-test, ***= *p* < 0.001

At last, chromatin states predicted to be associated with enhancer regions in PBMCs, available on ENCODE, were examined to see if any of these overlapped with the 4C-seq interactions. Thus, 16 4C-seq interactions from the type-I NF1 microdeletion patient pool and 15 interactions from the healthy donor pool that overlapped with enhancer states were identified. Patients and controls shared 6 interactions, while patients acquired 10 new interactions between 4C-seq viewpoint and enhancer regions and lost 9 of them, compared with controls (Supplementary Table S11). The new DNA-DNA interactions found in the deletion flanking region, also involving sequences associated with enhancer chromatin states, are suggestive of the establishment of a new regulatory context acting *in cis* on the type-I NF1 microdeletion flanking genes.

### Genetic mechanisms involved in phenotypic variability of NF1 microdeletion syndrome

#### Evaluation of variants possible leading to pseudo-dominance or with a modifier significance

To identify the affecting function variants associated with the clinical condition of 19 out of 22 enrolled type-I NF1 microdeletion patients, we performed a Target Resequencing analysis of genes mapped to 17q11.2 region, RAS pathway genes and neurofibromin interactor genes. We generated about 700 thousand reads per sample on average. After duplicate removal, we obtained an average of 688 thousand reads mapped on NCBI human reference genome (build GRCh37). The mean depth was 85X (ranging from 28 to 180), with more than 98% of the targeted regions covered by NGS reads in each sample (Supplementary Table S7). Only 420 variants passed all the filtering steps imposed by our pipeline (e.g. low-depth, strand-bias etc.). By means of Annovar, the annotation analysis revealed 416 single-nucleotide polymorphisms (SNPs) and 4 deletions (inDel) (Supplementary Table S12). The majority of SNPs occur within exonic regions (329), while the remaining are located in the 3′UTR (10), in 5′UTR or upstream (13), in intergenic regions (3), in intronics (60) and splicing (1) regions. Among the coding variants, 131 and 193 are missense and synonymous respectively (Supplementary Table S12). Aiming at selecting coding non-silent rare variants in 1000genomes database (1000g2015aug_eur), we applied a stringent filter, according to a MAF < 0.01, obtaining 49 variants in 38 genes. Furthermore, we applied a combinatorial approach based on different prediction tools to assess the possible effects of non-synonymous SNPs (snSNPs) and we kept only the variants predicted damaging by at least 11 tools out of 20, obtaining a total of 15 variants. One of them was excluded for the high frequency reported in ExAC (0.25) and gnomADv.2.1.1 (0.17) databases (Supplementary Table S13).

Using NGS we identified in ten out of 19 patients (53%) 14 constitutive rare SNVs, with four subjects, namely N22, N27, N75, and N80, carrying more than one variant (Table 3).

**Table 3:** Rare variants identified in type-I NF1 microdeletion patients

Patient ID	Gene	Exon	dbSNP build 154	Nucleotide variation	Protein variation		Clinical significance	Dampred
N22	*A2ML1 NM_001282424.2*	16	rs200964353	c.1796G > A	p.(Gly599Asp)	Likely benign		13.2
	*CPD NM_001199775.1*	19	rs1337539221	c.2929G > A	p.(Gly977Arg)	Uncertain		16.2
N26	*RNF135 NM_032322.4*	5	rs61749868	c.1245G > T	p.(Trp415Cys)	Likely benign		11.2
N27	*GAB2 NM_012296.3*	4	rs561641037	c.862 A > T	p.(Ile288Phe)	Uncertain		12.2
	*RASAL1 NM_001193521.1*	16	rs142556970	c.1804T > C	p.(Phe602Leu)	Likely Benign		11.2
N28	*PHF12 NM_001033561.2*	8	-	c.1246 C > G*	p.(His416Asp)	Uncertain		13.2
N43	*RAF1 NM_002880.3*	17	rs748925179	c.1811 C > G	p.(Ser604Cys)	Likely pathogenic		18.2
N75	*RASAL2 NM_170692.4*	6	-	c.1342 C > G*	p.(Gln448Glu)	Uncertain		13.2
	*RASA1 NM_002890.3*	20	rs1359038183	c.2656 C > T	p.(Pro886Ser)	Likely pathogenic		19.2
N76	*SARM1 NM_015077.4*	9	rs144613221	c.1498T > C	p.(Tyr500His)	Uncertain		13.16
N80	*LRP1 NM_002332.3*	51	rs962402779	c.8218G > A	p.(Glu2740Lys)	Uncertain		12.2
	*PAK4 NM_001014834.3*	3	-	c.449 A > G*	p.(Gln150Arg)	Uncertain		14.2
N82	*RASAL3 NM_022904.3*	13	-	c.1983G > A	p.(Met661Ile)	Uncertain		17.2
N83	*GAB2 NM_012296.3*	3	rs770269898	c.350 A > G	p.(Glu117Gly)	Likely Benign		11.2

*Novel variants, not reported in any of the consulted databases (see Table S13)

Dampred: Damage prediction score calculated by Annovar (range 1–20)

All the selected SNVs were heterozygous missense variants except for the hemizygous one affecting *RNF135*, a candidate gene for the pseudo-dominance mechanism, which maps within the type-I NF1 microdeletion interval. Three out of 14 variants (~ 29%) were novel (Table 3 and Supplementary Table S13) and inheritance was not available for any of the variants. Besides the SNV affecting *RNF135*, further three SNVs occurred in genes mapped at chromosome 17q, namely *CPD*, *SARM1*, and *PHF12*, which flanked the microdeletion centromerically. In addition, we identified ten SNVs in genes of the RAS pathway, i.e., *RASAL1*, *GAB2*, *RAF1*, *RASAL2*, *RASA1*, *LRP1*, *PAK4*, *RASAL3*, and *A2ML1*. We did not detect any variants in genes neither flanking the microdeletion telomerically nor enconding neurofibromin interactors.

To evaluate possible pseudo-dominance/modifying mechanisms related to the selected variants, we assessed the clinical significance according to the ACMG/AMP criteria (see “Material and methods” section), which led to their classification into three groups (Table 3 and Supplementary Table S13): (1) “Likely benign” (four out of 14 SNVs, 29%); (2) “Uncertain” (8/14, 57%); (3) “Likely pathogenic” (2/14, 14%). Specifically, the most promising variants are the amino acid substitutions c.1811 C > G (p.(Ser604Cys)) and c.2656 C > T (p.(Pro886Ser)) affecting the *RAF1* and *RASA1* genes, respectively. Both SNVs have been so far reported just once in gnomAD v.2.1.1 database and they are not included in DECIPHER, the RASA1: c.2656 C > T (p.(Pro886Ser)) is reported in the ClinVar database with uncertain significance in a patient with Capillary malformation-arteriovenous malformation syndrome (Supplementary Table S13).

### In silico prediction of protein stability of RAF1 and RASA1 selected variants

*In silico* prediction of protein stability and conformational changes have been performed (see Supplementary file SF1 for details) for mutated RAF1 p.(Ser604Cys) and RASA1 p.(Pro886Ser) gene products, classified as likely pathogenic based on our criteria.

*In silico* analysis of *RAF1* c.1811 C > G (p.(Ser604Cys)) showed that the residue substitution localizes in the protein core in the kinase domain Cr3, which is responsible for maintaining of RAF1 in its inactive form, and is a highly conserved residue across species. The amino acid change from serine, a small polar amino acid to cysteine, a hydrophobic amino acid that forms disulfide bonds, could affect the protein functionality. This is confirmed by prediction tools (see Supplementary file SF1 for the details), which indicate the destabilizing effect of the mutation (ΔΔG: -0.308 kcal/mol). In the mutant protein the interatomic interactions are altered compared to those of the wild-type protein (Fig. 5 6 Panel b and c). The mutation p.(Ser604Cys) could also affect the interaction with the tyrosine-protein kinase FYN (Final ΔΔG = 1.467).

**Fig. 6:**
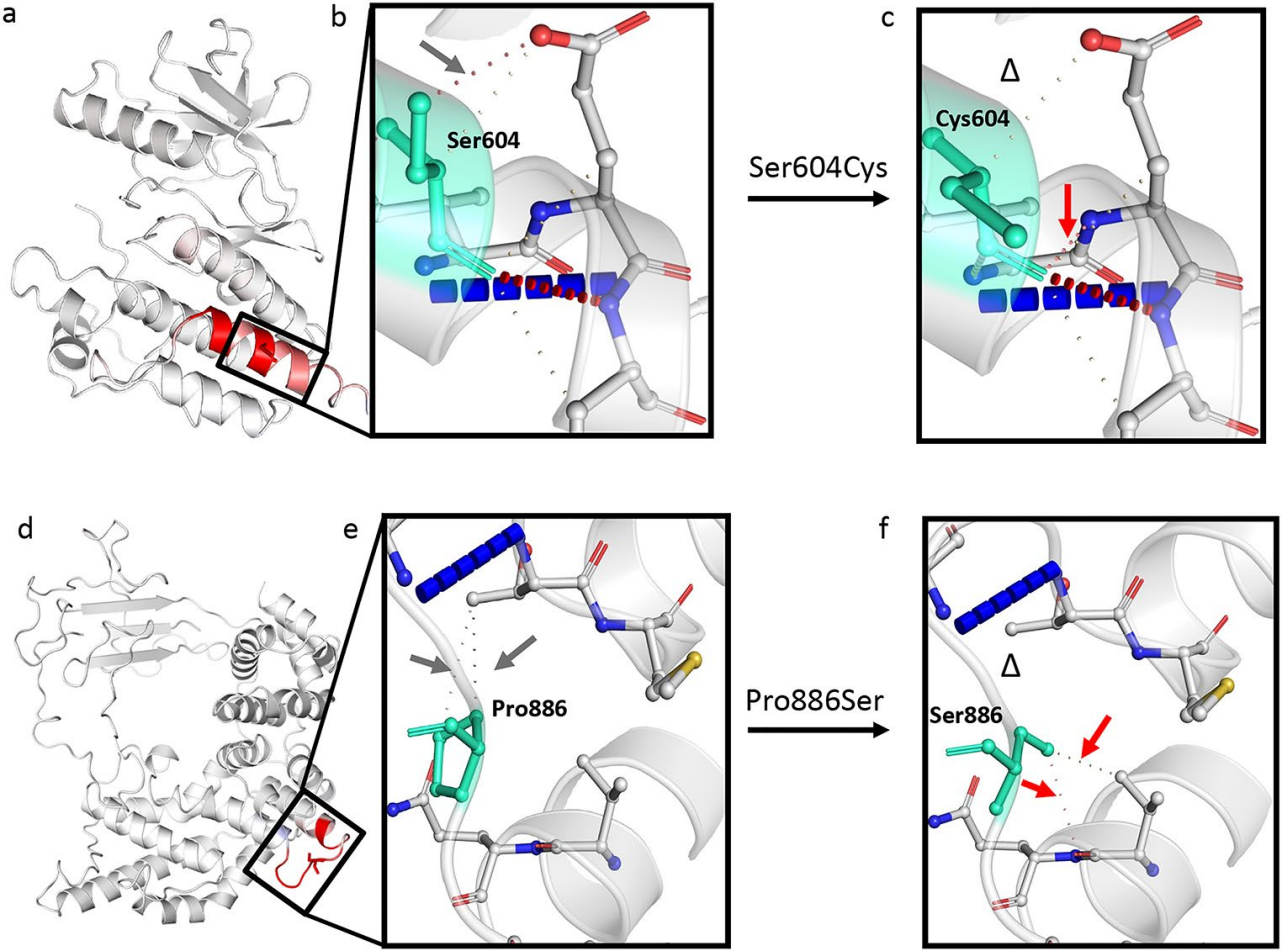
Models of wild-type and mutated RAF1 and RASA1 proteins obtained with the DynaMut tool. (**a**) Enzyme modelling structure of the mutated RAF1 p.(Ser604Cys) protein. Amino acids are colored according to the change in vibrational entropy upon mutation. The red color represents a gain in flexibility; (**b**) Prediction of interatomic interactions due to the wild-type Ser residue at position 604. The grey arrow indicates a water-mediated hydrogen bond that is disrupted in the mutant protein (panel c); (**c**) Prediction of interatomic interactions due to substitution of the Ser residue of 604nd by Cys. The mutant protein loses a water-mediated hydrogen bond (Δ) and gains a new one (red arrow). The mutation is destabilizing: ΔΔG: -0.370 kcal/mol. Color legend: hydrogen bond in dark red colour dotted lines, water-mediated hydrogen bond in light red dotted lines, ionic bonds in grey dotted lines, halogen bonds in dark blue dotted lines, the residues at position 604 in light green; (**d**) Enzyme modelling structure of mutated RASA1 p.(Pro886Ser) protein. Amino acids are colored according to the vibrational entropy change upon mutation. The red color represents a gain in flexibility; (**e**) Prediction of interatomic interactions due to the wild-type Pro residue at position 886. The grey arrows indicate two ionic bonds that are disrupted in the mutant protein (panel f); (**f**) Prediction of interatomic interactions due to substitution of the Pro residue of 886nd by Ser. Two ionic bonds (Δ) are disrupted due to mutation. One new ionic bond (red arrow) and one new water-mediated hydrogen bond (red arrow) are formed in the mutant. The effect of the mutation is to stabilize ΔΔG: 0.019 kcal/mol. Color legend: water-mediated hydrogen bond in light red dotted lines, ionic bonds in grey dotted lines, halogen bonds in dark blue dotted lines, the residues at position 886 in light green

*In silico* analysis of *RASA1* c.2656 C > T (p.(Pro886Ser)) showed that the residue substitution localizes in the protein core in the RAS-GTPase Activating Protein (RAS-GAP) domain, responsible for the GTPase activity of the protein, and it is a highly conserved residue through species. The amino acid change from proline, a small hydrophobic amino acid to serin, a small polar amino acid could affect the protein functionality. The distinctive cyclic structure of the proline side chain gives it a greater conformational rigidity as compared to other amino acids.

Proline 886 is near the beginning of an α-helix and can act as a perturbator in secondary structure elements. The prediction tools (see Supplementary SF1 for the details) indicate a slightly stabilizing effect of the mutation (ΔΔG: 0.019 kcal/mol). The prediction was made on the best generated model (see Supplementary SF1) that covers the protein region of interest, but with only the 25.29% of sequence identity with RASA1 entire protein. In the mutant protein the interatomic interactions are altered compared to those of the wild-type protein. (Fig. 5, 6 Panels e and f).

## Discussion

Type-I NF1 microdeletion mainly results from a germline NAHR, unlike the other classes of NF1 microdeletion that arise from recombination events that also occur at post-zygotic level (reviewed by Kehrer-Sawatzki et al. 2017). Mosaicism can be involved in expression variability of the clinical phenotype in a fraction of NF1 microdeletion patients, particularly those with type-II and atypical NF1 deletions. In contrast, type-I NF1 microdeletion are predominantly germline deletions. It is still unclear whether the clinical phenotype associated with type-I NF1 microdeletion is mainly caused by the haploinsufficiency of the genes located within the type-I NF1 microdeletion interval or whether additional pathogenetic mechanisms contribute to the clinical expression. To investigate the NF1 microdeletion syndrome etiopathogenesis, we enrolled 22 type-I NF1 microdeletion syndrome patients. The incidence of the clinical signs, included in the classical NF1 phenotype or the typical ones of the microdeletion syndrome, in our cohort is comparable to that reported in the literature (reviewed by Kehrer-Sawatzki et al. 2017), allowing us to consider our case studies as sufficiently representative of the clinical picture of type-I NF1 microdeletion syndrome.

Position effect on expression of genes flanking deletions has been poor studied in microdeletion syndromes and has only been described in one NF1 microdeletion syndrome patient (Ferrari et al. 2017; Tritto et al. 2023b). We performed *in silico* analysis of 17q11.2 TADs on a single lymphoblastoid cell line, in accordance with the subsequent real time analysis on retrotranscribed cDNA derived from patient whole blood RNA. Nevertheless, 3D Genome Browser shows that the chromatin topological organization of 17q11.2 region is fairly conserved in other cell lines, such as NHEK (normal human epidermal keratinocytes), DLPFC (dorsolateral prefrontal cortex) and GZ (germinal zone of human cerebral cortex) (Supplementary Fig. S4). Type-I NF1 microdeletion syndrome patients, losing the same TADs, should be subjected to the expression dysregulation of the same genes that should be involved in the onset of common abnormal clinical traits.

We demonstrated the position effect in peripheral blood analyzing genes ubiquitously expressed, therefore their expression is possibly affected in other tissues, even if the regulatory elements may vary in cells derived from other tissues. We found an upregulation of *GOSR1*, *CPD*, *NSRP1*, and *PHF12* genes and a downregulation of *RHOT1*, *c17orf75*, and *ZNF207* genes, never correlated to NF1 microdeletion syndrome, at our knowledge. Of note, three out of the four over-expressed genes belong to the partially deleted TAD, upstream of the deletion, and the three hypo-expressed genes map to the first telomeric TAD following the deletion, leading us to speculate that underlying the position effect may be a mechanism of enhancer competition (Kleinjan and van Heyningen 1998) which is restricted to the new putative TAD, presumably generated as a result of the deletion. Genetic alterations that change the architecture of TADs, resulting in non-canonical enhancer-gene interactions that increase the expression of the interacting genes and lead to decreased expression of genes they normally regulate, causing severe phenotypes, have been reported previously. Lupiáñez and colleagues described how heterozygous deletions on chromosome 2q35-36 that alter the higher-order chromatin organization of the *WNT6*/*IHH*/*EPHA4*/*PAX3* locus can allow interactions between a cluster of limb enhancers, normally associated with *EPHA4*, and the *PAX3* promoter region, leading to an ectopic limb expression of *PAX3* and causing brachydactyly (Lupiáñez et al. 2015).

The limitation of *in silico* analysis of TADs is that they may vary based on the different methods of identification (Dang et al. 2023), but our results are supported by the deregulation of flanking genes expression, which is restricted to the two partially deleted TADs, with the exception of one gene.

Given that gene expression regulation is a complex biological process, resulting from the activity of several mechanisms, a different modulation of gene activity due to the position effect might impact the penetrance or severity degree of a specific clinical trait shared by type-I NF1 microdeletion patients. Notably, there may be correlations between the expression deregulation of the *RHOT1* and *ZNF207* genes and some phenotypic features observed in type-I NF1 microdeletion syndrome patients. *RHOT1* maps 70 Kb far from the telomeric breakpoint of type-I NF1 microdeletion. *RHOT1* encodes MIRO1, a protein belonging to the Rho GTPases and reported to be involved in mitochondrial transport (Fransson et al. 2003) and in migration and polarity of lymphocytes (Morlino et al. 2014). MIRO1 is known to interfere with mitochondrial quality control and Mitochondria-ER contact sites (MERCS). Low expression levels of RhoT1 appear to be significantly associated with lymph node metastasis and shorter survival in pancreatic cancer patients. Interestingly, pancreatic endocrine tumors occur in patients with NF1 and have been reported to be a cause of death in these patients (Khosrotehrani et al. 2003; Jensen et al. 2008). Deregulation of *RHOT1* expression may be a risk factor for pancreatic cancer in NF1 microdeletion patients, although the reported data refer to NF1 patients without distinguishing between different forms of NF1. MERCs are known to be involved in the regulation of many cellular functions (calcium homeostasis, lipid metabolism, autophagy, and apoptosis) with a broad implication into synaptic events, due to the presence of these contacts in various parts of neurons and glial cells. Despite MERCs function is not fully explored, it is intriguing to observe a downregulation of a gene strictly related to MERCs in this peculiar class of patients, with a typical neurodevelopmental involvement (Shirokova et al. 2020). *ZNF207* is located 300 Kb distant from the telomeric breakpoint of type-I NF1 microdeletion. This gene encodes for BuGZ, a zinc finger protein that binds to spindle microtubules and regulates chromosome alignment during mitosis (Jiang et al. 2014). SiRNA-mediated knockdown of *ZNF207*, carried out on human embryonic stem cells (hESCs), has shown that reduction of its gene expression impairs self-renewal and pluripo(Finn et al. 2019)tency of these cells by blocking ectoderm differentiation (Fang et al. 2018). Because nervous tissue and epidermis, both widely affected by the symptoms of NF1 microdeletion syndrome, derive from the ectoderm, hypo-expression of *ZNF207* could be a candidate mechanism for playing a role in the altered development of these tissues, although further investigation is needed to confirm this hypothesis.

To identify the putative chromatin remodeling causative of the position effect found in microdeletion patients, a 4C-Seq assay was carried out on samples from three type-I NF1 microdeletion patients and three controls. The analysis was performed on the two pools of patients and healthy donors to minimize the biological variability, possibly due to cellular and allelic differences (Finn et al. 2019). Interactions between the selected viewpoint, represented by the *RHOT1* promoter located after the telomeric deletion breakpoint, and the whole genome, which differed in patients and controls, confirmed an altered chromatin folding of the chromosomal region 17q11.2 in our patients.

Interestingly, in the NF1 microdeletion patients, the gain of a new interaction between the viewpoint and the *SLC6A4* gene, mapped to the centromeric TAD partially deleted and found to be hyper-expressed by qPCR in the peripheral blood of the 15 patients, was identified. This evidence further suggests that the deletion may lead to the formation of a new TAD, given by the fusion between the centromeric and telomeric TADs, which may be responsible for the change in the regulatory landscape acting *in cis* on genes showing aberrant expression.

The loss of interactions between the viewpoint and the sequences contained in the chromosome region within the deletion shown by the 4C-seq data revealed that chromatin remodeling occurs not only in the region flanking the deletion, but also in the deletion interval, on the non-deleted homologous chromosome. This evidence suggests that the presence of the deletion may lead to a change in the chromatin structural organization of the homologous chromosome, indicating the involvement of trans-acting mechanisms. This hypothesis is supported by the higher expression of some genes within the deletion interval in our microdeletion patients compared to the expected 50% reduction.

Furthermore, the loss of DNA-DNA interactions within the type-I NF1 deletion leads us to speculate on a possible crosstalk between the two chromosomes. Although to our knowledge the occurrence of this molecular mechanism as a result of a microdeletion has not been previously described, some evidence supports the hypothesis of crosstalk between homologous chromosomes, even during the interphase of the cell cycle. The transcription machinery tends to co-express the two alleles of the same gene, located on the two homologous chromosomes, almost simultaneously (Santoni et al. 2017). In addition, chromosomes preferentially occupy defined regions in the cell nucleus, which are known as chromosomal territories, which represent an additional level of gene expression regulation (Bolzer et al. 2005). Changes in the three-dimensional conformation of specific chromosomes and the area of their chromosomal territories, due to chromosomal rearrangements, have been observed in the interphase DNA of spermatozoa, by two-dimensional fluorescent in situ hybridization (FISH) performed both in spermatozoa with normal chromosome arrangement and in those characterized by chromosomal imbalance (Mebrek et al. 2020). The results obtained from Mebrek’s study demonstrate that nuclear architecture has a fragile organization and that chromosomal abnormalities can affect the entire nucleus. Understanding the molecular basis leading to the chromatin remodeling, observed on the non-deleted chromosome in patients with type-I NF1 microdeletion, may allow the identification of a pathogenetic mechanism common to other microdeletion syndromes.

Most of the genes included in the type-I NF1 microdeletion interval have been identified as dosage-sensitive genes in previous studies (reviewed by Kehrer-Sawatzki et al. 2017). By checking the constraint metric probability of being LoF intolerant (pLI), according to gnomAD v.2.1.1 database, the 14 deleted genes could be classified into three categories: (1) LoF intolerant, characterized by a pLI ≥ 0.9 (5/14, 36%), including the *ATAD5*, *NF1*, *OMG*, *RAB11FIP4*, and *SUZ12* genes, whose haploinsufficiency could lead to pathological consequences in patients with type-I *NF1* microdeletion, thus worsening their phenotype compared to that of NF1 patients without the microdeletion; (2) likely haplosufficient genes with 0.1 < pLI < 0.9 (2/14, 14%), including *COPRS* and *TEFM*; (3) LoF tolerant genes with a pLI ≤ 0.1 (7/14, 50%), namely, *ADAP2*, *CRLF3*, *EVI2A*, *EVI2B*, *LRRC37B*, *RNF135*, and *UTP6*, for which the pLI metric suggests the existence of a different pathomechanism, e.g., a two-hit recessive model, in contributing to the patients’ complex phenotype (Supplementary Fig. S5 and Table S2). Consistently, deletion of *ATAD5, NF1, RAB11FIP4*, *SUZ12*, and *COPRS* genes n to may increase tumor risk in NF1 microdeletion patients (as reported by Kehrer-Sawatzki et al. 2017), while *OMG* deletion gene has been previously associated to cognitive impairment in this NF1 patients’ subgroup (Venturin 2004). Heterozygous deletion of the *TEFM* gene, whose product acts as a general elongation factor for mtDNA transcription in mammalian mitochondria (Minczuk et al. 2011), does not impair mitochondrial function of mtDNA replication and transcription (Minczuk et al. 2011), suggesting that its hemizygosity is unlike affecting the phenotype of NF1 microdeletion patients. Of note, among the genes predicted to be LoF tolerant, *ADAP2* and *RNF135* were found to be associated to cardiovascular malformations, overgrowth, and dysmorphisms by functional studies (Douglas et al. 2007; Venturin et al. 2014). These findings suggest that the hemizygosity contribution of these two genes to the NF1 microdeletion syndrome phenotype should be considered, as facial dysmorphism is a trait shared by most of the NF1 microdeletion patients.

While haploinsufficiency of genes within the deletion interval and position effect on expression regulation of genes flanking the deletion are phenomena shared by patients with the same deletion and expected to be associated to the onset of common clinical traits, the pathomechanism of the occurrence of rare clinical features of the disease remains an unresolved problem (Mautner et al. 2010). Presence of hypomorphic or recessive LoF variants is expected in human genome of healthy individuals (MacArthur et al. 2012; Deltas 2018). Their co-occurrence with the “pathogenic mutation” could aggravate the clinical phenotype as genetic modifiers and the peculiar traits in type-I NF1 microdeletion patients should be related to genetic variation. Typically, these variants belong to the same pathway or to the same interactome of the full mutation are present in the normal population with a low MAF due to negative constraints, to avoid a detrimental epistatic effect resulting from co-occurrence of more hypomorphic variants (Deltas 2018).

Because variants in more than one gene of the same pathway can contribute the severity of a specific phenotype (Löwik et al. 2008; Li et al. 2020), on the basis of the recently reported finding on a RASopathy (Ferrari et al. 2020; Tritto et al. 2023a), we investigate a possible addictive effect of RAS pathway gene variants leading to identify potential modifier genes. The targeted NGS analysis of RAS pathway and neurofibromin interactor genes allowed us to identify likely pathogenic variants within two RAS pathway genes in the following patients: N75 (*RASA1)* and N43 (*RAF1*).

The *RASA1* c.2656 C > T (p.(Pro886Ser) variant was found in N75, a ten years’ old patient, with an early diagnosis (at 8 months of age), based on the presence of CALs and lentigo, who, at 4 years of age, developed a brainstem glioma that remained radiologically stable and clinically asymptomatic. His phenotype was also characterized by congenital hypothyroidism and facial hemangiomas, both of which are uncommon in NF1 phenotype. In addition, the clinical course was complicated by delayed psychomotor development and brainstem glioma diagnosed at 4 years of age. The NF1 tumor predisposing syndrome increases the risk of developing brain tumors, which are observed in approximately 15–20% of NF1 cases (Seminog and Goldacre 2013; Uusitalo et al. 2016). The optic pathway glioma, a pilocytic astrocytoma, is observed in about 15% of children with NF1, while non-optic gliomas with different histological subtypes including high-grade glioma develop more frequently at older ages (Sellmer et al. 2017). Taking into account the biological function of RASA1 and the disease related to its pathogenic variants, we propose that the severe expression of the clinical phenotype of this patient could be modulated by the p.(Pro886Ser) variant.

*RASA1* variants have been associated with vascular malformation syndromes characterized by hereditary capillary malformations (CM) with or without arteriovenous malformations (AVM), arteriovenous fistulas (AVF), or Parkes Weber syndrome; however, the phenotypic spectrum of the constitutional variants of *RASA1* is still being defined (Wooderchak-Donahue et al. 2018). Therefore, it is possible to hypothesize that the *RASA1* variant in our patient may be responsible for the facial hemangiomas. This hypothesis is supported by the report in the ClinVar database of a patient with CM-AVM carrying the same *RASA1* variant. The child also developed a brainstem glioma, which is reported in 10% of NF1 children with a median age of onset of about 7 years and an indolent course in most cases (Costa and Gutmann 2020). To our knowledge, no genotype-phenotype correlation studies have been reported in non-optic gliomas in patients with NF1. However, since *RASA1* encodes an effector of the RAS pathway involved in carcinogenesis, its role in tumor etiopathogenesis in the patient cannot be excluded. In particular, the *RASA1* mutation is predicted to be deleterious and involves the protein core in the RAS-GAP domain, which is responsible for the GTPase activity of the protein and for the inactivation of the RAS pathway. Mutations in additional RAS pathway genes with a similar function to neurofibromin may trigger a similar tumorigenic process leading to glioma onset. Alterations in the binding sites of either RASA1 or RAS p21 proteins are associated with basal cell carcinomas. In addition, the co-deletion of *RASA1* and *NF1* results in the development of T-cell acute lymphoblastic leukemia (Lubeck et al. 2015).

Likewise, a possible modulatory effect can be postulated for p.(Ser604Cys) variant in *RAF1*. In our cohort, the p.(Ser604Cys) variant in the *RAF1* gene was found in N43, a 47 years-old patient with a common microdeletion NF1 phenotype, including ID, dysmorphic features, and cerebrovascular pathology, who was diagnosed with thyroid C-Cell Hyperplasia. The clinical picture of this patient is burdened by the developmental signs typical of NF1 microdeletion syndrome (i.e. ID and dysmorphic features) and it is possible to speculate that her features could be modulated by this variant in *RAF1*, which is one of the RAS pathway genes, potentially amplifying the RAS pathway dysregulation itself caused by the NF1 microdeletion. The p.(Ser604Cys) variant is a potentially damaging *RAF1* mutation in the Cr3 domain, which is involved in the maintenance of RAF1 in its inactive conformation by interacting with the 14-3-3 protein family members dimers (YIP-SCHNEIDER et al. 2000). Of note, the mutation p.(Leu603Pro), adjacent to p.(Ser604Cys), observed in a Noonan patient, showed impaired kinase activity and reduced ERK activation as did a truncated RAF1 protein p.(Arg254fs) (Dhandapany et al. 2014). Pathogenic *RAF1* mutations in the Cr2 domain causing Noonan syndrome are mainly associated to the onset of cardiovascular malformations, although sporadic cases of cerebrovascular abnormalities have been reported (Zarate et al. 2014; Hartill et al. 2017). The state of knowledge of its role in vasculopathy brain changes is less consolidated but there are evidences in *Raf1* homozygous knock out mouse endothelial cells (Wimmer et al. 2012) of the direct critical role of *Raf1* in angiogenesis. Even though full expression of microdeletion type-I phenotype and cerebrovascular abnormalities were also detected in other patients of our cohort in whom we did not find variants possibly associated with this specific clinical sign, it could be hypothesized that an effect of the p.(Ser604Cys) variant contributes to N43 patient phenotype. In this view, the occurrence of hypomorphic variants in RAS pathway genes, co-present with the main disease-mutation, could bias the clinical phenotype of microdeletion patients.

The reported genetic conditions in the above two patients and the associated variants are suggestive that peculiar phenotypes could possibly be determined by hypomorphic variants modulating the clinical picture in terms of variability and/or severity of the NF1 microdeletion syndrome. Variants in RAS pathway effectors that may function as genetic modifiers could exacerbate typical NF1 clinical features such as the neurofibroma burden or congenital abnormalities common to other RASopathies.

Regarding the pseudo-dominance effect, we identified a variant in the *RNF135* gene in patient N26 who presented with learning problems, overgrowth, and coarse sections. The c.1245G > T (p.(Trp415Cys) variant in the *RNF135* gene is located in the B30.2/SPRY domain. The variant is rare (MAF < 0.01) and has been previously described in three patients with Sotos syndrome features of uncertain significance (Visser et al. 2012) and reported in GnomAD also in homozygosity, thus its pathogenic significance remains to be verified.

Besides the complex effects generated by NF1 microdeletions on deregulation of multiple molecular pathways, it appears that the NF1 haploinsufficiency per se, in addition to RAS hyperactivation, directly or indirectly causes a deregulation of genes encoding neurofibromin interactors such as syndecans. It has recently been reported that syndecan *SDC2*, *SDC3* and *SDC4* were found to be hyper-expressed in both NF1 and Spinal Neurofibromatosis (SNF) patients (Bettinaglio et al. 2023). This complex molecular landscape is difficult to elucidate. The current challenge is to unravel the perturbation of homeostasis of different pathways related to neurofibromin functions, addressing the identification of possible druggable genes.

The clinical information reported in our patients lacks precise standardization, regarding the ID and the real tumor burden evaluation, because the current guidelines of Regione Lombardia do not require that all NF1 patients undergo cognitive tests or whole-body MRIs. Nevertheless, the integrated genomic approach used in this study sheds light on the heterogeneity of the pathogenetic mechanisms involved in determining the clinical characteristics and variability of this genetic disorder.

NF1 deletion syndrome is a complex disease, burdened by the variability of severe medical complications; a better knowledge of the pathogenesis underlying the clinical variability is mandatory to hypothesize future designs of personalized prevention programs for potentially life-threatening complications.

## Conclusions

Our findings strongly suggest that, in addition to the known effects of the widely studied haploinsufficient genes, other mechanisms should be considered to understand the etiopathogenesis of NF1 microdeletion syndrome. We found that specific clinical traits, such as cutaneous and neurodevelopmental abnormalities or cancer susceptibility, could be associated with the position effect of *ZNF207* and *RHOT1* downregulation, respectively. However, other possible correlations can be evaluated. These results will promote studies on larger cohort to establish the transcriptomic profile associated with position effect. Furthermore, the generation of differentiated derived-iPSCs cell lines from NF1 microdeletion patients will allow to investigate the position effect of this deletion in different tissues, also addressing the identification of possible pharmacological targets for effective treatments. This is a pilot study that will highlight different approaches that will help to address the phenotype-genotype-epigenotype correlations not only in NF1 microdeletion syndrome, but more generally in genetic syndromes based on the involvement of wide genomic regions. The associated application of NGS should provide new insights into genetic signatures, leading to the identification of potential genetic modifiers inherited as hypomorphic variants from normal parents and potentially involved in the variable expressivity of a disease. This should improve prognosis and appropriate follow-up with a positive relapse in terms of patient management and social costs.

### Electronic supplementary material

Below is the link to the electronic supplementary material.

Supplementary Material 1

Supplementary Material 2

Supplementary Material 3

Supplementary Material 4

Supplementary Material 5

Supplementary Material 6

Supplementary Material 7

Supplementary Material 8

Supplementary Material 9

Supplementary Material 10

Supplementary Material 11

Supplementary Material 12

Supplementary Material 13

Supplementary Material 14

Supplementary Material 15

Supplementary Material 16

Supplementary Material 17

Supplementary Material 18

Supplementary Material 19

Supplementary Material 20

## Data Availability

All the selected variants were described according to recommendations of the Human Genome Structural Variant (HGSV) consortium. Raw reads of NGS data are available in NCBI Short-read Archive (SRA, https://www.ncbi.nlm.nih.gov/sra/) under the accession number PRJNA688415. Raw reads of 4C-seq data have been deposited under the accession number PRJNA688415. However, they will be released after publication.
