# Supplementary material for: Genetic/epigenetic effects in NF1 microdeletion syndrome: beyond the haploinsufficiency, looking at the contribution of not deleted genes
Source: Hum Genet. 2024 Jun 14;143(6):775–95. doi: 10.1007/s00439-024-02683-0 (PMC11186880; doi:10.1007/s00439-024-02683-0)
Supplement: Supplementary file 1 — Supplementary Material 1 [file 439_2024_2683_MOESM1_ESM.docx]

**FS1. Prediction method of protein changes upon mutations**

Due to lack in the PDB protein database (<https://www.rcsb.org/>) of the crystal structure of the N terminal part of RASA1 protein, the protein model of RASA1 was obtained through the “User Template mode” tool of Swiss database (<https://swissmodel.expasy.org/interactive#structure>) by submitting the Uniprot (<https://www.uniprot.org/>) ID of RASA1 ([P20936](https://www.uniprot.org/uniprot/P20936)). The model obtained by Swiss for RASA1, residue interval 588-1028, was based on C2-GAP fragment of rat synGAP (PDB Swiss ID: 3bxj), which displays a sequence identity of 25.29% with modelled sequence and covers the residue interval 588-1028. The Serine/threonine-protein kinase C-raf RAF1 crystal structure was retrieved from PDB protein database (<https://www.rcsb.org/>) with the ID:3OMV and cover the residues 323-618 of the entire RAF1 protein. The pdb files of Raf1 and Rasa1 were submitted to Dynamut tool (<http://biosig.unimelb.edu.au/dynamut/>) for the prediction of protein stability changes and conformational changes upon RAF1 p.Ser604Cys and RASA1 p.Pro886Ser mutations.

The Elaspic tool (<http://elaspic.kimlab.org/>) was used to predict the stability change in the interaction between RAF1 p.Ser604Cys mutated protein and FYN protein.
