# Supplementary figures and images for: Genetic/epigenetic effects in NF1 microdeletion syndrome: beyond the haploinsufficiency, looking at the contribution of not deleted genes
Source: Hum Genet. 2024 Jun 14;143(6):775–95. doi: 10.1007/s00439-024-02683-0 (PMC11186880; doi:10.1007/s00439-024-02683-0)

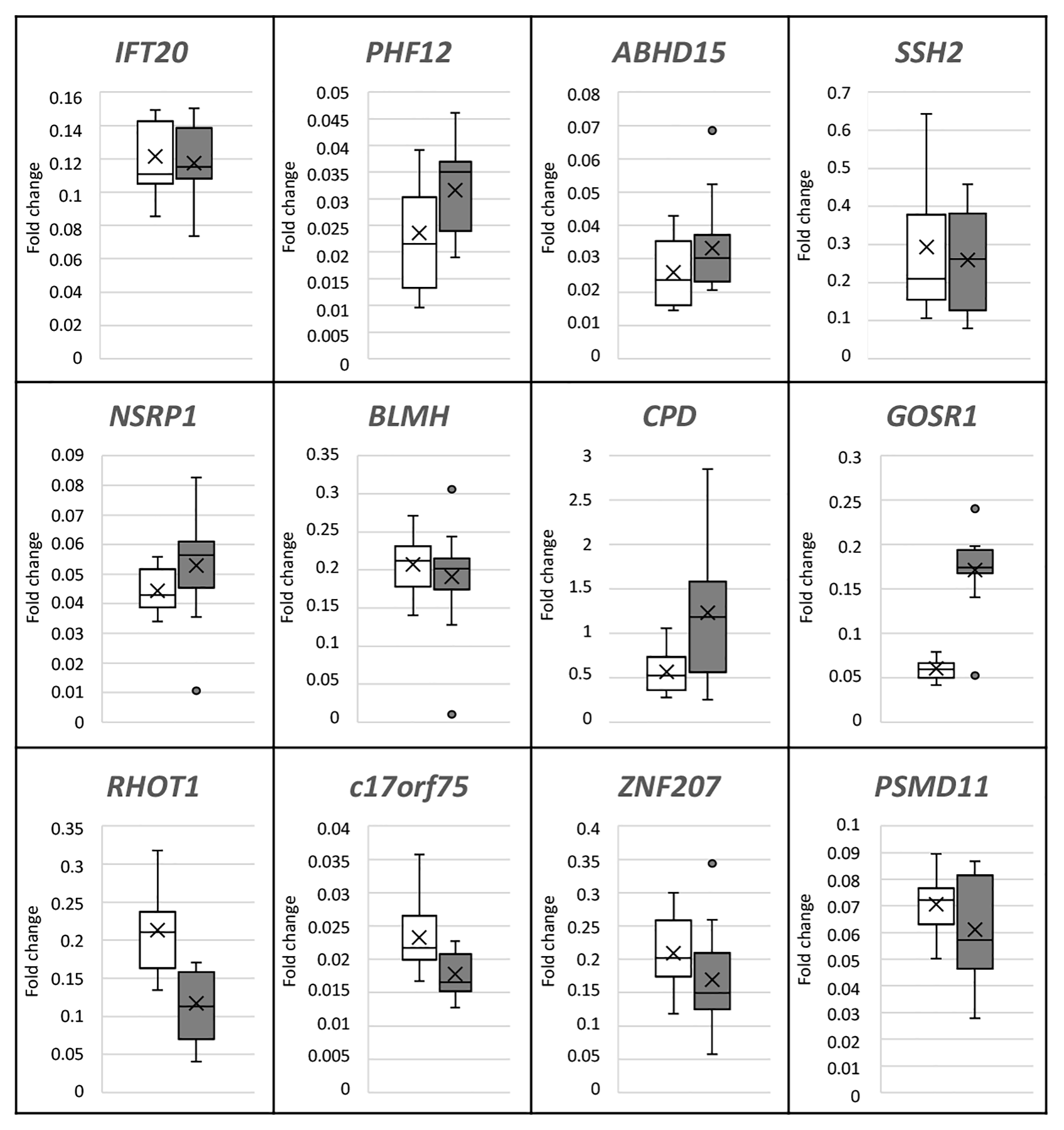

Supplement: Supplementary file 2 — Supplementary Material 2 [file 439_2024_2683_MOESM2_ESM.tif]

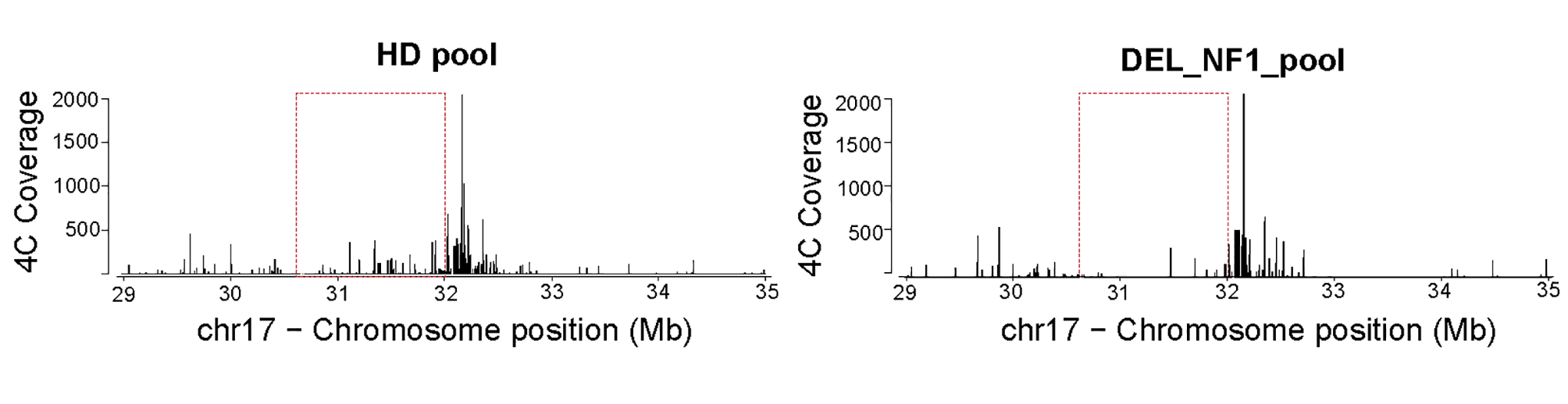

Supplement: Supplementary file 3 — Supplementary Material 3 [file 439_2024_2683_MOESM3_ESM.tif]

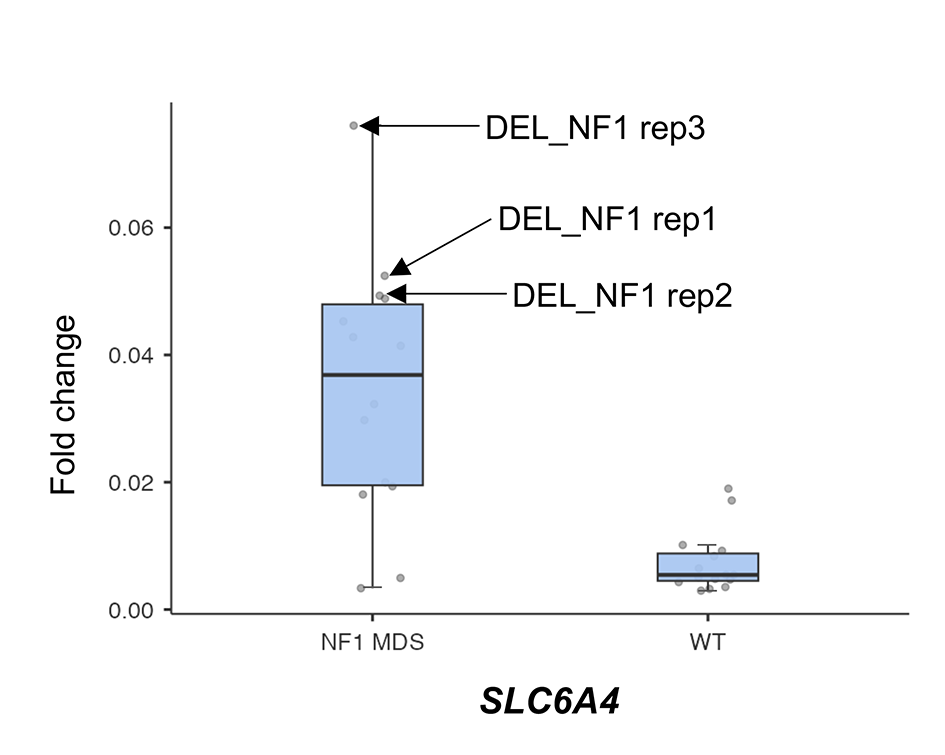

Supplement: Supplementary file 4 — Supplementary Material 4 [file 439_2024_2683_MOESM4_ESM.tif]

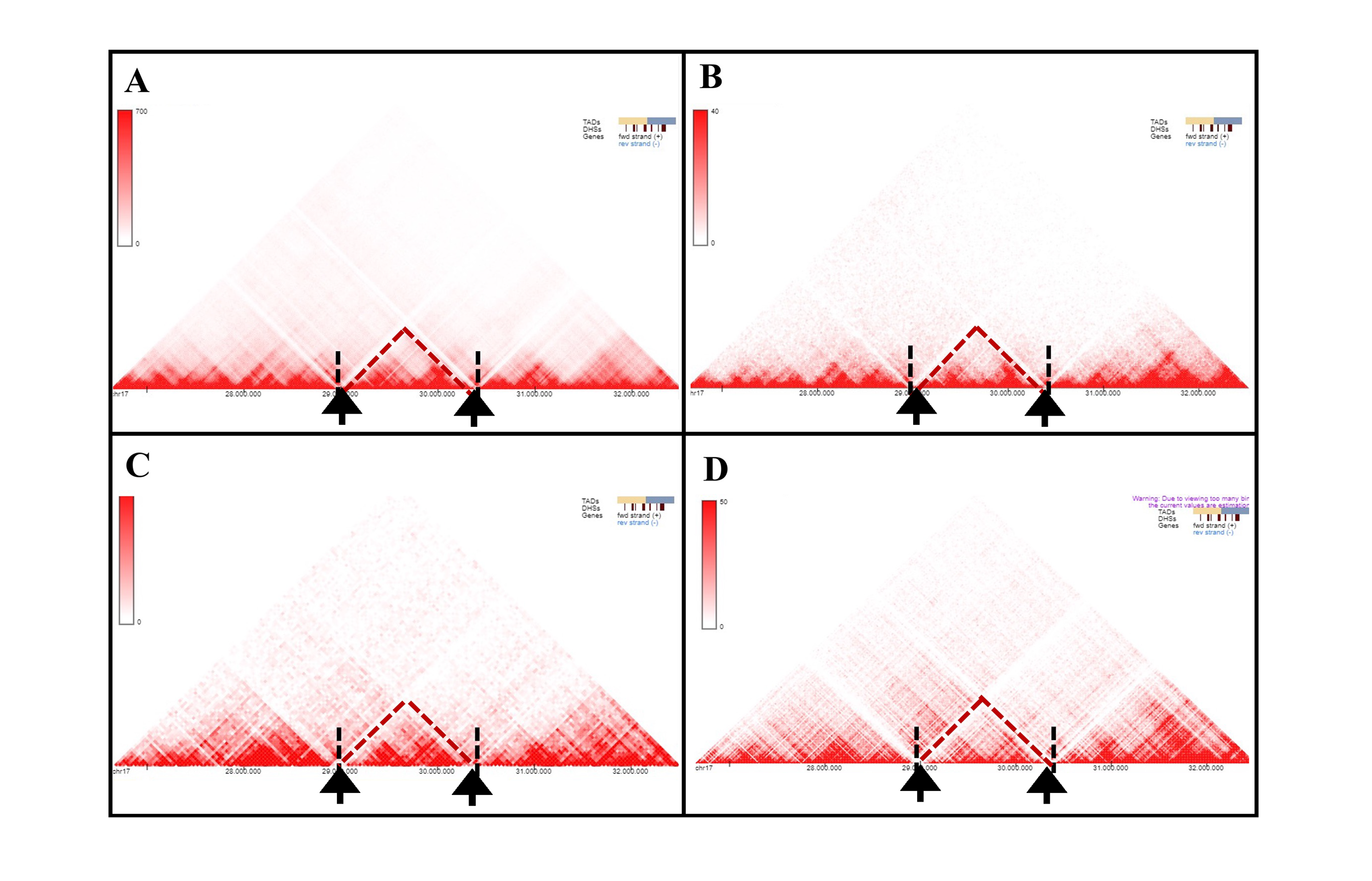

Supplement: Supplementary file 5 — Supplementary Material 5 [file 439_2024_2683_MOESM5_ESM.tif]

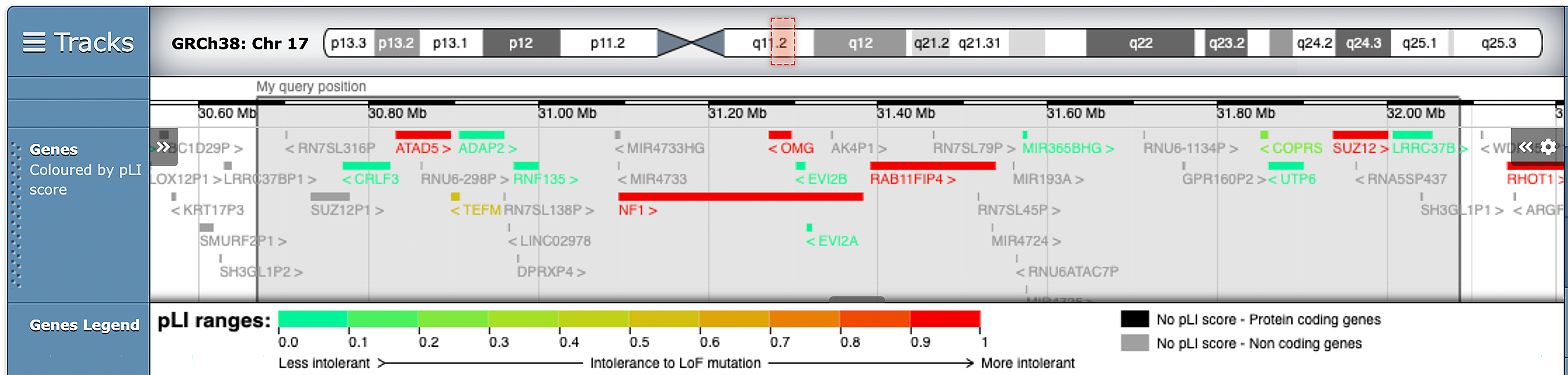

Supplement: Supplementary file 6 — Supplementary Material 6 [file 439_2024_2683_MOESM6_ESM.tif]
