# Supplementary material for: Genetic/epigenetic effects in NF1 microdeletion syndrome: beyond the haploinsufficiency, looking at the contribution of not deleted genes
Source: Hum Genet. 2024 Jun 14;143(6):775–95. doi: 10.1007/s00439-024-02683-0 (PMC11186880; doi:10.1007/s00439-024-02683-0)
Supplement: Supplementary file 7 — Supplementary Material 7 [file 439_2024_2683_MOESM7_ESM.docx]

**Figure S1.** Distribution of expression levels of the 17q11.2 selected genes in NF1 microdeletion patients and controls. The box plots show the distribution of the quantitative expression levels (2^-ΔCt^) of twelve 17q11.2 selected genes analyzed by qPCR, in the peripheral blood of 15 patients with type-I NF1 microdeletion syndrome (shown in gray) and 15 wild-type controls (shown in white). The bottom and the top of the boxes represent the first quartile (25th percentile) and the third quartile (75th percentile), respectively. The line in the middle of the box is the median (50th percentile). The whiskers show the minimum and maximum value of the distribution, excluding the outliers, represented as spots outside of the boxes.

**Figure S2.** Coverage profiles of samples analyzed by 4C-seq assay. Coverage profiles derived by collecting the samples into two pools, one consisting of three healthy donors (HD pool) and the other one including three patients with type-I NF1 microdeletion (DEL_NF1_pool). The graphs show on the y-axis the 4C coverage along the genomic region of chromosome 17 included in the 3 Mb upstream to and downstream of the viewpoint, whose genomic coordinates are indicated in Mb on the x-axis (build Human GRCh38/hg38).

**Figure S3.** Distribution of expression levels of the *SLC6A4* gene in NF1 microdeletion patients and controls. The box plots show the distribution of the quantitative expression levels (2^-ΔCt^) of *SLC6A4* gene analyzed by qPCR, in the peripheral blood of 15 patients with type-I NF1 microdeletion syndrome and 15 wild-type controls. The bottom and the top of the boxes represent the first quartile (25th percentile) and the third quartile (75th percentile), respectively. The line in the middle of the box is the median (50th percentile). The whiskers show the minimum and maximum value of the distribution, excluding the outliers, represented as spots outside of the boxes. The dots correspond to the individual samples analyzed, of which those corresponding to the patients also analyzed by 4C-seq were indicated (DEL_NF1 rep1, DEL_NF1 rep2, DEL_NF1 rep3).

**Figure S4.** Visualization of the 17q11.2 topologically associating domains in different cell lines. The heatmaps obtained from 3D Genome Browser show the TADs mapping in the 17q11.2 region, in accordance with the Hi-C data in (A) GM12878 lymphoblastoid cells, (B) NHEK (normal human epidermal keratinocytes), (C) DLPFC (dorsolateral prefrontal cortex), and (D) GZ (germinal zone of human cerebral cortex). The region involved by the type-I NF1 microdeletion (whose breakpoints are indicated by the vertical dashed lines) maintains a fairly similar chromatin topological organization (dark red dashed triangle) in these four cell lines, with two extended genomic boundaries between TADs removed by the deletion (black arrows).

**Figure S5.** Genes included in the type-I NF1 microdeletion interval with relative pLI score. The screen obtained from Decipher Genome Browser shows that among the 14 protein-coding genes involved by the type-I NF1 microdeletion (highlighted by the gray box), five genes (in red) characterized by a pLI ≥0.9 could be classified as LoF intolerant, and seven genes (in bright teal) with a pLI ≤0.1 as LoF tolerant. The last two genes with 0.1<pLI<0.9 (in green and yellow) could be likely haplosufficient genes.
